# Ecological niche modeling of *Astragalusmembranaceus* var. *mongholicus* medicinal plants in Inner Mongolia, China

**DOI:** 10.1038/s41598-020-69391-3

**Published:** 2020-07-27

**Authors:** Min Yang, Ziyan Li, Lanbo Liu, Agula Bo, Chunhong Zhang, Minhui Li

**Affiliations:** 1 Department of Pharmacy, Baotou Medical College, Baotou, 014060 China; 2 Baotou Meteorological Bureau of Inner Mongolia Autonomous Region, Baotou, 014030 China; 3 Inner Mongolia Key Laboratory of Chinese Medicinal Materials Resource, Baotou Medical College, Baotou, 014060 China; 4 Pharmaceutical Laboratory, Inner Mongolia Institute of Traditional Chinese Medicine, Hohhot, 010020 China; 5 Guangxi Key Laboratory of Medicinal Resources Protection and Genetic Improvement, Guangxi Botanical Garden of Medicinal Plants, Nanning, 530023 China; 6 Inner Mongolia Key Laboratory of Characteristic Geoherbs Resources Protection and Utilization, Baotou Medical College, Baotou, 014060 China

**Keywords:** Conservation biology, Ecological modelling, Quality control, Plant breeding, Plant domestication, Plant ecology

## Abstract

Radix Astragali is commonly used in traditional Chinese medicine, and its quality is closely related to ecological factors, such as climate and soil, in the production area. To provide high-quality Radix Astragali to Chinese and foreign markets, we used maximum entropy model and statistical analysis method, combined with data on ecological factors, *Astragalus* *membranaceus* var. *mongholicus* geographical distribution, and index component content to predict the ecological suitability distribution of *A.* *membranaceus* var. *mongholicus* and establish the relationship between astragaloside IV and calycosin-7-glucoside in this species and ecological factors. Subsequently, we could determine the suitability regionalization of high-quality *A.* *membranaceus* var. *mongholicus* in Inner Mongolia, China. The results showed that the standard deviation of seasonal changes in temperature (40.6%), precipitation in October (15.7%), vegetation type (14.3%), soil type (9.2%), and mean sunshine duration in the growing season (9.1%) were the top five most influential factors out of the 17 main ecological factors affecting the distribution of *A.* *membranaceus* var. *mongholicus*. The standard deviation of seasonal changes in temperature, precipitation in October, precipitation in April, soil pH, and mean sunshine duration in the growing season were found to be the key ecological factors affecting the accumulation of astragaloside IV and calycosin-7-glucoside in *A.* *membranaceus* var. *mongholicus*. The regions with the highest-quality *A.* *membranaceus* var. *mongholicus* were distributed in Baotou (Guyang County), Hohhot (Wuchuan County), and central Wulanchabu (Chahar Right Middle Banner, Chahar Right Back Banner, and Shangdu County) and its surroundings in Inner Mongolia. Baotou, Hohhot, and their surrounding areas were the main traditional production areas of *A.* *membranaceus* var. *mongholicus*, and central Wulanchabu was a potentially suitable distribution area of this species*.* The main production areas were consistent with the actual production base of *A.* *membranaceus* var. *mongholicus*. This study therefore provides a scientific basis to guide the cultivation of *A.* *membranaceus* var. *mongholicus*.

## Introduction

**Figure 1:**
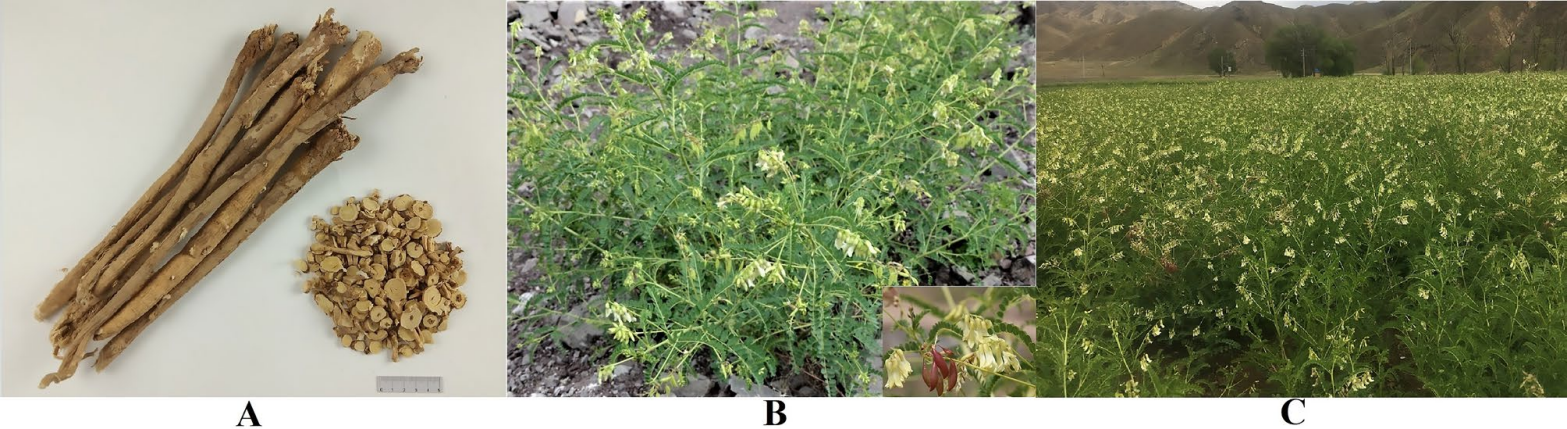
Image of Radix Astragali (**A**), the wild *A.* *membranaceus* var. *mongholicus* (**B**) and cultivated *A.* *membranaceus* var. *mongholicus* (**C**).

Radix Astragali is a well-known and widely used traditional Chinese medicine (Fig. 1A) and is classified as the top grade in *Shen* *Nong's* *Materia* *Medica* (Shen Nong Ben Cao Jing). It possesses tonic, hepatoprotective, diuretic, and expectorant properties and is used to clinically treat cardiovascular, immunological, respiratory, and hepatic diseases^1,2^. The *Chinese* *Pharmacopoeia* (2015 edition) lists 204 species of patented traditional Chinese medicines containing Radix Astragali, accounting for approximately 14% of the total number of traditional medicines^3^. In addition, Radix Astragali is a vital ingredient in health products, plays a key role in the food and cosmetic industries, and can strengthen the body’s immune system and promote youthfulness when used over a long period of time. Owing to its medicinal effects and health value, the demand for Radix Astragali in both Chinese and foreign markets has increased. Radix Astragali is exported by China, and 35 countries and regions, including South Korea, Hong Kong, Japan, the United States, Thailand, Singapore, Indonesia, and Australia are involved in its trade^4,5^.

*Astragalus* *membranaceus* (Fisch.) Bunge var. *mongholicus* (Bunge) P.K. Hsiao (Leguminosae) is the main source of Radix Astragali (Fig. 1B,C). This species naturally occurs mainly in Inner Mongolia, Shanxi, Gansu, Hebei, and Ningxia Province in China^6^. Inner Mongolia is currently one of the main growing regions of *A.* *membranaceus* var. *mongholicus*, and Radix Astragali from this area is well-known globally because of its thick and long main roots, few lateral roots, soft texture, sufficient powder, strong medicinal properties, and high quality^7^. With the increasing scarcity of wild resources, *A.* *membranaceus* var. *mongholicus* has been included in the “China Rare Endangered Plant Directory”^8^. At present, Radix Astragali from cultivated species is the leading product in the Chinese market. The quality of *A.* *membranaceus* var. *mongholicus* produced in different regions varies greatly and is mainly affected by the natural environment, cultivation techniques, growth years, harvesting, and processing^9–12^. Indeed, the impact of soil, topography, climate, and other ecological factors on the quality of this medicinal plant cannot be ignored^13,14^.

The Inner Mongolia Autonomous Region is located in northern China (37° 24′–53° 23′ N; 97° 12′–126° 04′ E). Its terrain covers plateaus, mountains, hills, plains, deserts, rivers, and lakes. The climate is dominated by a temperate zone continental monsoon climate transitioning from humid to arid from east to west; this area is thus characterized by complex terrain and climatic factors as well as great changes in the ecological environment^15^. In recent decades, the cultivation of *A.* *membranaceus* var. *mongholicus* in Inner Mongolia has developed rapidly. However, some planting companies and famers have ignored the impact of the ecological environment on the quality of this herb and have chosen cultivation sites blindly, possibly compromising the quality of cultivated *A.* *membranaceus* var. *mongholicus*^16^. Based on a fuller understanding of the regional characteristics and difference law of ecological factors in Inner Mongolia, this paper studied the ecological suitability regionalization and quality regionalization for *A.* *membranaceus* var. *mongholicus* in Inner Mongolia.

Recent technological advancements in Geographic Information Systems (GISs), remote sensing, decision support systems, and web-based applications have allowed for a powerful, precise, and sustainable intervention in agriculture in terms of where the farming should be done, and which crop will be the most suitable^17^. A wide range of methods have been developed including, but not limited to the analytical hierarchy process, ordered weighted averaging, the GIS-based logic scoring of preference, and machine learning, which provides the possibility of suitable planting areas for the medicinal plants^18^. The maximum entropy (Maxent) model, a machine learning method, is the most commonly used species distribution model with the advantages of simple and fast operation, and reasonable prediction results^19–21^. Traditional methods only use factors derived from expert opinion, such as supply, demand, and production, to analyze ecological suitability distribution and cultivation regions of medicinal plants. On the contrary, Maxent greatly reduces the influence of subjective factors, and therefore, it is widely used to predict the potentially suitable distribution regions for plants and even animals^22–26^.

**Figure 2:**
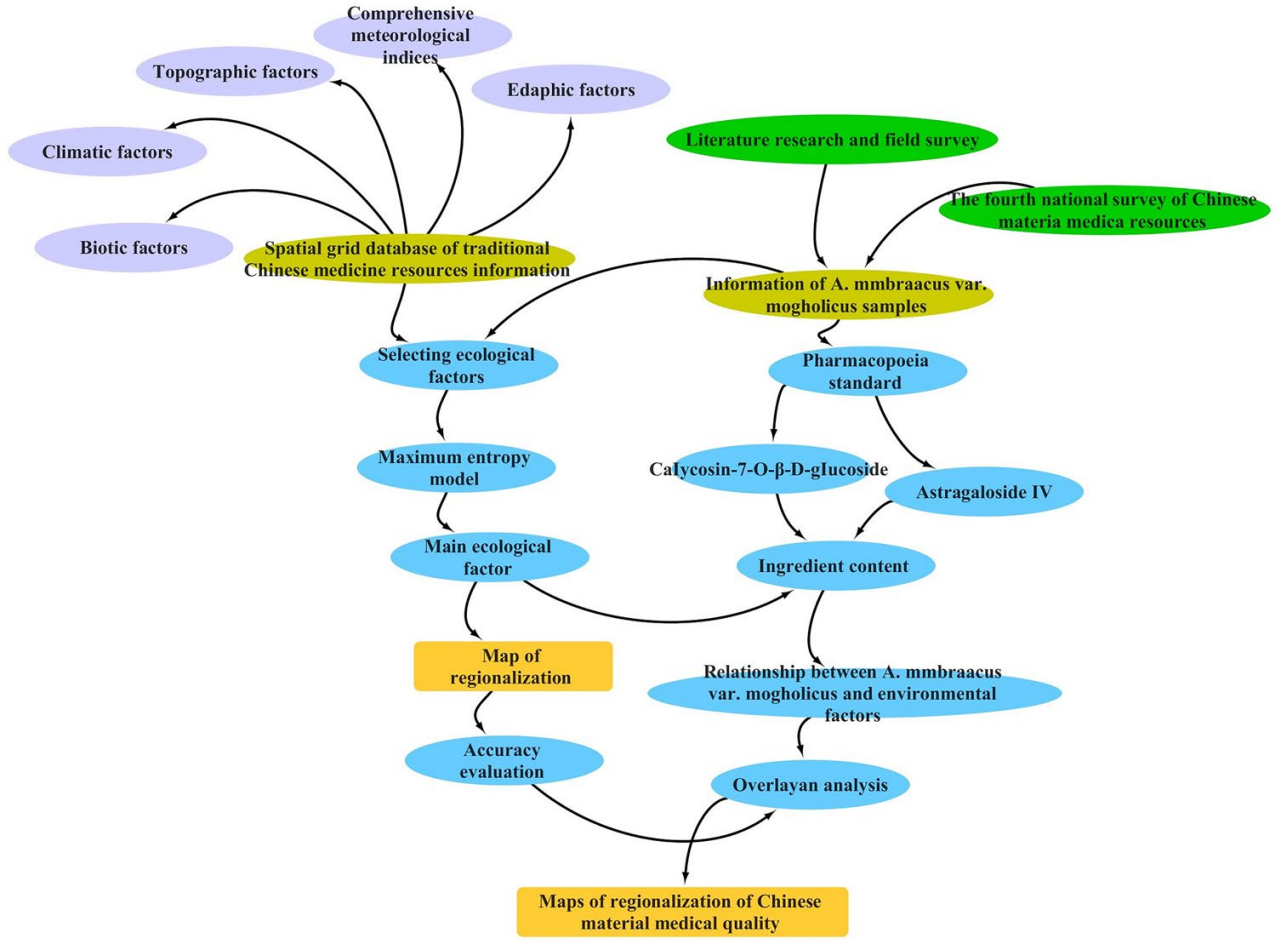
Mind map of the study on the ecological suitability and quality regionalization of *A.* *membranaceus* var. *mongholicus* based on maximum entropy modeling, in which purple represents the category of ecological factors, green represents the investigation method of *A.* *membranaceus* var. *mongholicus*, blue represents the selection of indicators and data processing, and yellow represents the data acquisition results and the final regionalization study results.

In this study, we used the MaxEnt model to predict the potentially suitable distribution area of *A.* *membranaceus* var. *mongholicus*, investigated the main environmental factors affecting its distribution, and analyzed them with the index components (astragaloside IV and calycosin-7-glucoside) representing the quality of *A.* *membranaceus* var. *mongholicus*. Finally, we combined the obtained results with ArcGIS to obtain areas that provide the best growth and quality of *A.* *membranaceus* var. *mongholicus* in Inner Mongolia, to provide a scientific basis for the study of cultivation and production site selection, and production regionalization. The obtained results could be used to guide production to ensure the production of the highest-quality Radix Astragali for both Chinese and foreign markets. Furthermore, the results provide a foundation for studying the value chain of Radix Astragali (high-value raw materials). Our recommendations in terms of the ecological suitability and quality regionalization of *A.* *membranaceus* var. *mongholicus* based MaxEnt modeling are depicted in Fig. 2.

## Results

### Preliminary screening of ecological factors

**Figure 3:**
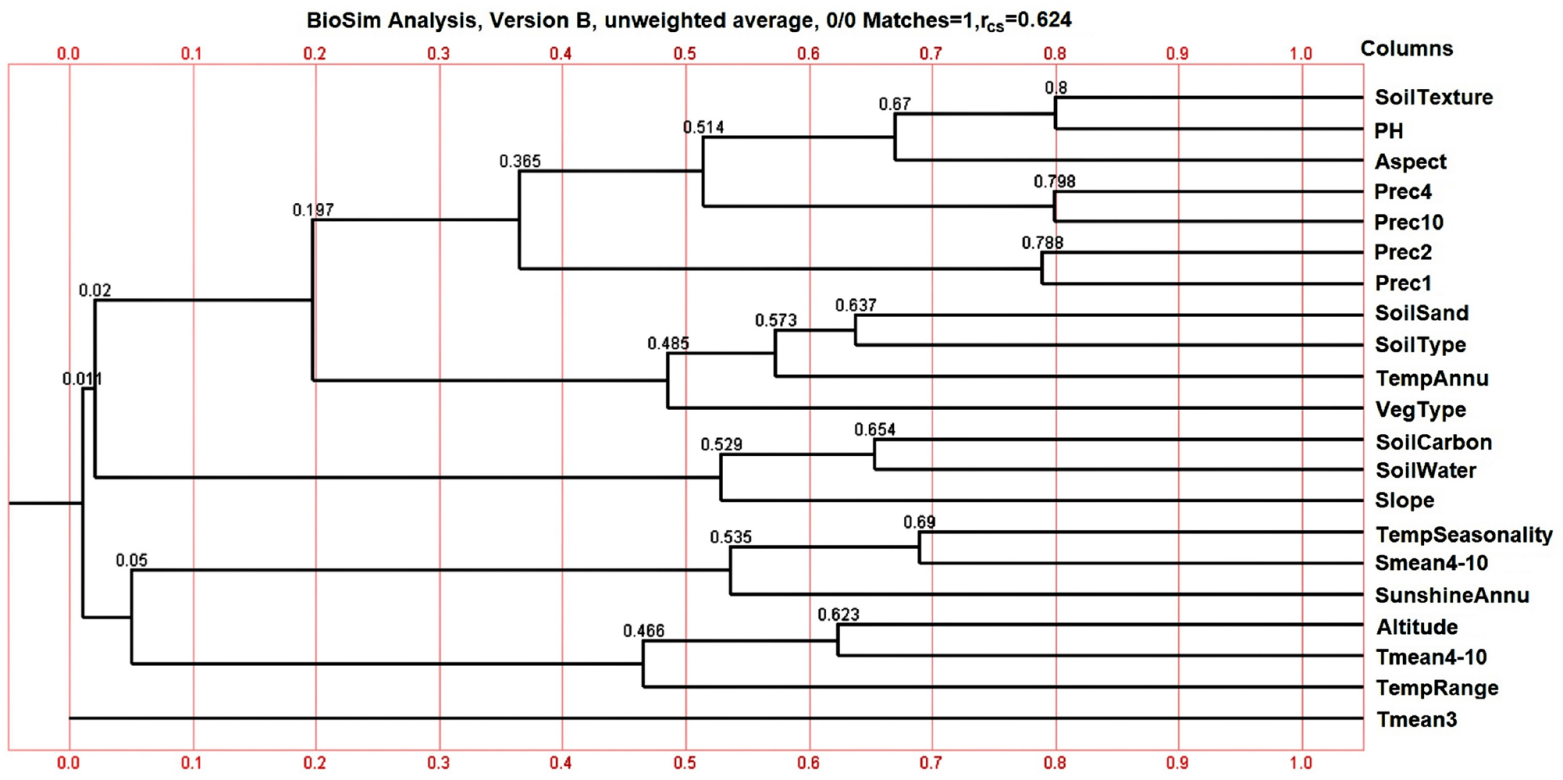
21 ecological factors with correlation coefficient ≤ 0.8 were preliminarily screened by BioSim2 software.

After performing five screenings using the Biosim2 software, 53 of 74 ecological factors were omitted from the analysis (Appendix 1 in Supplementary Information 1), and 21 ecological factors with correlation coefficients of ≤ 0.8 were retained after preliminary screening (Fig. 3), namely SoilTexture, pH, aspect, mean precipitation in January, February, April, and October (Prec1, 2, 4, and 10), SoilSand, SoilType, mean annual temperature (TempAnnu), vegetation type (VegType), SoilCarbon, SoilWater, slope, standard deviation of the seasonal changes in temperature (TempSeasonality), Smean4-10, SunshineAnnu, altitude, Tmean4-10, range of mean annual temperature (TempRange), mean temperature in March (Tmean3).

### Accuracy of MaxEnt model

**Figure 4:**
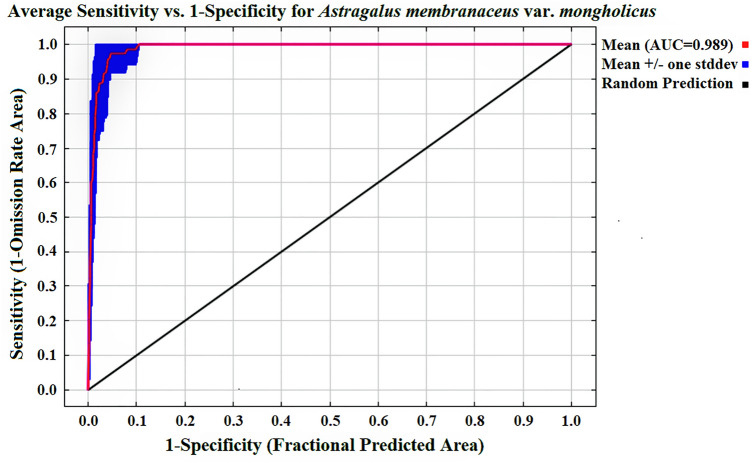
The mean AUC of the test samples of *A.* *membranaceus* var. *mongholicus* after ten calculations of the Maxent model.

Table 1 provides the estimates of the relative contributions of the environmental variables to the Maxent model. After 10 reiterations of the model was complete, the mean AUC of the *A.* *membranaceus* var. *mongholicus* test samples was estimated to be 0.989, and the standard deviation was 0.007, thereby indicating that the model was efficient in terms of its prediction capability, and therefore, the accuracy of its prediction results can be guaranteed (Fig. 4).

**Table 1:** The AUC value of Maxent model after ten replicated operations.

Project	1	2	3	4	5	6	7	8	9	10	Age
Training data	0.994	0.994	0.994	0.994	0.994	0.994	0.994	0.995	0.994	0.994	0.994
test data	0.986	0.997	0.990	0.990	0.995	0.984	0.990	0.970	0.991	0.992	0.989

### Ecological suitability regionalization of *A. membranaceus* var. *mongholicus* in Inner Mongolia

**Figure 5:**
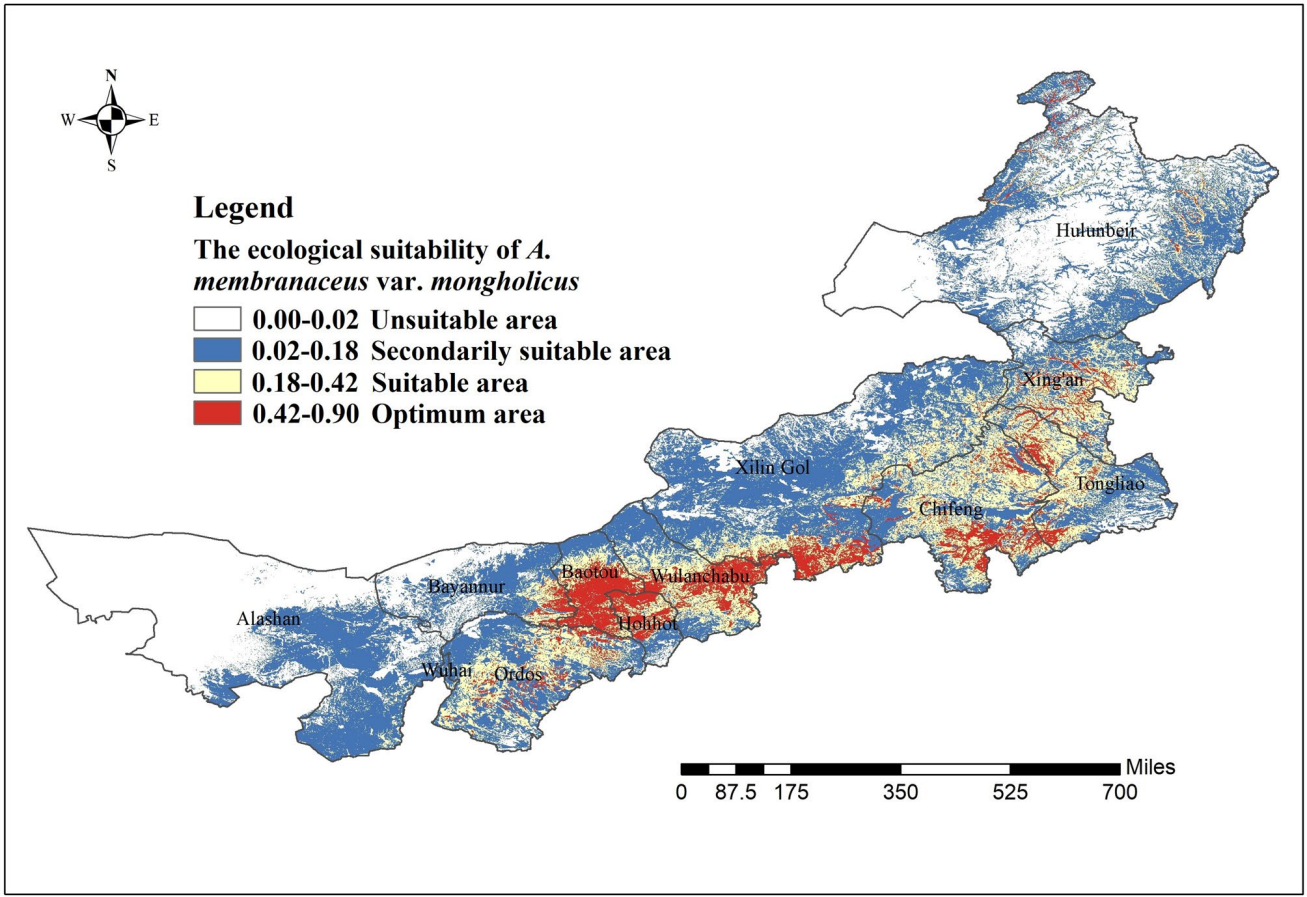
Regionalization maps of ecological suitability of *A.* *membranaceus* var. *mongholicus* in the Leagues and Cities. Generated using the ArcMap version 10.5 software (ESRI Inc., California, USA. https://www.esri.com/).

As depicted in Fig. 5, the most suitable areas for *A.* *membranaceus* var. *mongholicus* distribution were southeast Bayannur, northeast Ordos, central and southern Baotou and Wulanchabu City, central and northern Hohhot, southern Xilingol League, southern Chifeng City, western Tongliao City, and central Xingan League. The ecological suitability of *A.* *membranaceus* var. *mongholicus* in other areas in Inner Mongolia is detailed in Appendix 2 in Supplementary Information 1. The results show that the most suitable areas of *A.* *membranaceus* var. *mongholicus* covered 7.67% of the total area of Inner Mongolia. Huhhot (43.23%), Wulanchabu (36.06%), Baotou (20.35%), and Chifeng (18.24%) cities were determined as the main suitable producing areas. The total area and percentage of optimum area, suitable area, and secondarily suitable area in the Leagues or Cities in Inner Mongolia are detailed in Table 2.

**Table 2:** Suitabile area for *A.* *membranaceus* var. *mongholicus* distribution under various administrative levels.

Leagues or cities	Total area (km^2^)	Optimum area		Suitable area		Secondarily suitable area	
		Area (km^2^)	Percentage (%)	Area (km^2^)	Percentage (%)	Area (km^2^)	Percentage (%)
Bayannur League	65,135.08	3,373.23	5.18	5,026.24	7.72	27,910.95	42.85
Baotou City	27,889.94	5,474.67	20.35	6,995.39	25.08	14,839.72	53.21
Wulanchabu City	54,280.41	19,572.31	36.06	17,865.11	32.91	15,525.56	28.60
Chifeng City	87,353.27	15,930.28	18.24	40,437.60	46.29	29,242.13	33.48
Tongliao City	58,947.05	6,931.52	11.74	22,043.90	37.40	22,822.72	38.72
Ordos City	87,061.67	8,083.03	9.20	30,299.56	34.80	42,744.99	49.10
Huhhot City	17,031.10	7,362.79	43.23	3,786.03	22.23	3,951.39	23.20
Xing'an League	54,706.07	6,068.60	11.09	22,533.05	41.19	21,293.88	38.92
Xilin Gol	199,526.19	11,969.12	6.00	32,131.30	16.10	117,752.88	59.02
Hulunbeir City	253,561.97	3,061.58	1.21	15,869.45	6.26	89,498.01	35.30
Alashan	239,542.23	0.67	0	1946.54	0.81	78,414.26	32.74
Wuhai City	1532.02	9.99	0.65	242.93	15.86	1,243.70	81.18

In Inner Mongolia, Guyang County in Baotou City, Wuchuan County in Hohhot City, and their surrounding areas are traditional *A.* *membranaceus* var. *mongholicus* production areas, and large wild populations of the species were once distributed in these areas. This species is being cultivated in these areas since the 1970s, and with the increased demand, the production areas have continuously expanded^27^. At present, Guyang County, Wuchuan County, Harqin Banner in Chifeng City and Urad Front Banner in Bayannaoer City encompass the main cultivation areas of *A.* *membranaceus* var. *mongholicus* in Inner Mongolia, and Xingshunxi Town and Xiashihao Town in Guyang County contain many good agricultural practices (GAP) bases of this species. The results of ecological suitability regionalization of *A.* *membranaceus* var. *mongholicus* in Inner Mongolia were consistent with its actual production. Furthermore, in addition to the traditional production and main cultivation areas of the species, according to the ecological suitability regionalization results, Urad Middle Banner in Bayannur City; Darhan Muminggan Joint Banner, Tumd Right Banner in Baotou City; Siziwang Banner, Chahar Right Front, Middle, and Back Banners, Xinghe, Shangdu, and Huade Counties in Wulanchabu City; Bairin Right and Left Banners, Ar Horqin Banner, Ongniud Banner, and Aohan Banner in Chifeng City; Jarud Banner and Naiman Banner in Tongliao City; Dalad Banner in Ordos City; Tumd Left Banner, Togtoh County, and Horinger County in Hohhot City; Taibus Banner, Duolun County, Xianghuang Banner, Zhengxiangbai Banner and Zhenglan Banner in Xinlin Gol, a small part of central Xing’an League; and northern and eastern Hulunbeir are the potential distribution areas of *A.* *membranaceus* var. *mongholicus*.

### Main ecological factors affecting *A. membranaceus* var. *mongholicus* growth

**Figure 6:**
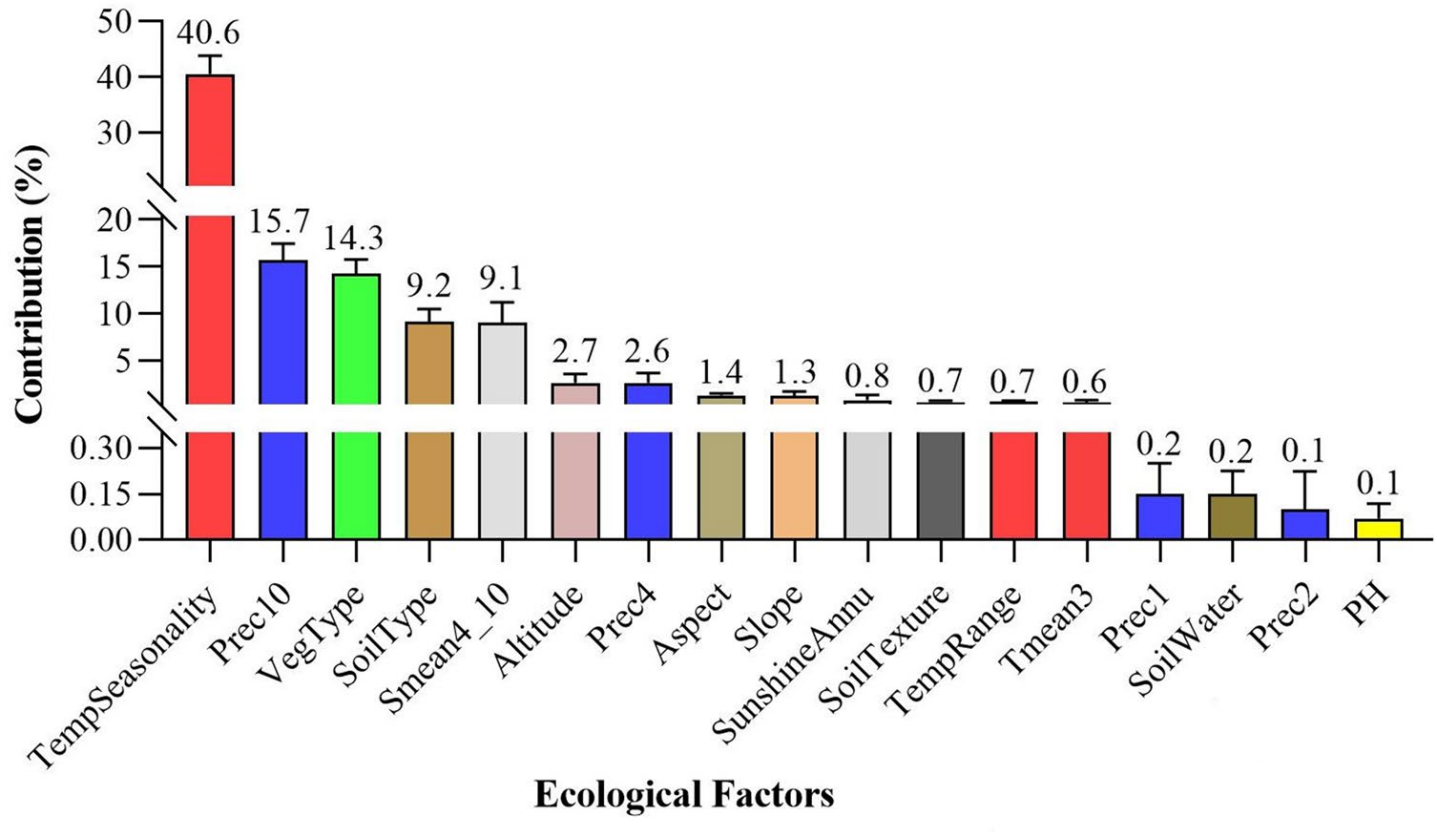
The contribution rate of each ecological factor to the growth of *A.* *membranaceus* var. *mongholicus.*

**Figure 7:**
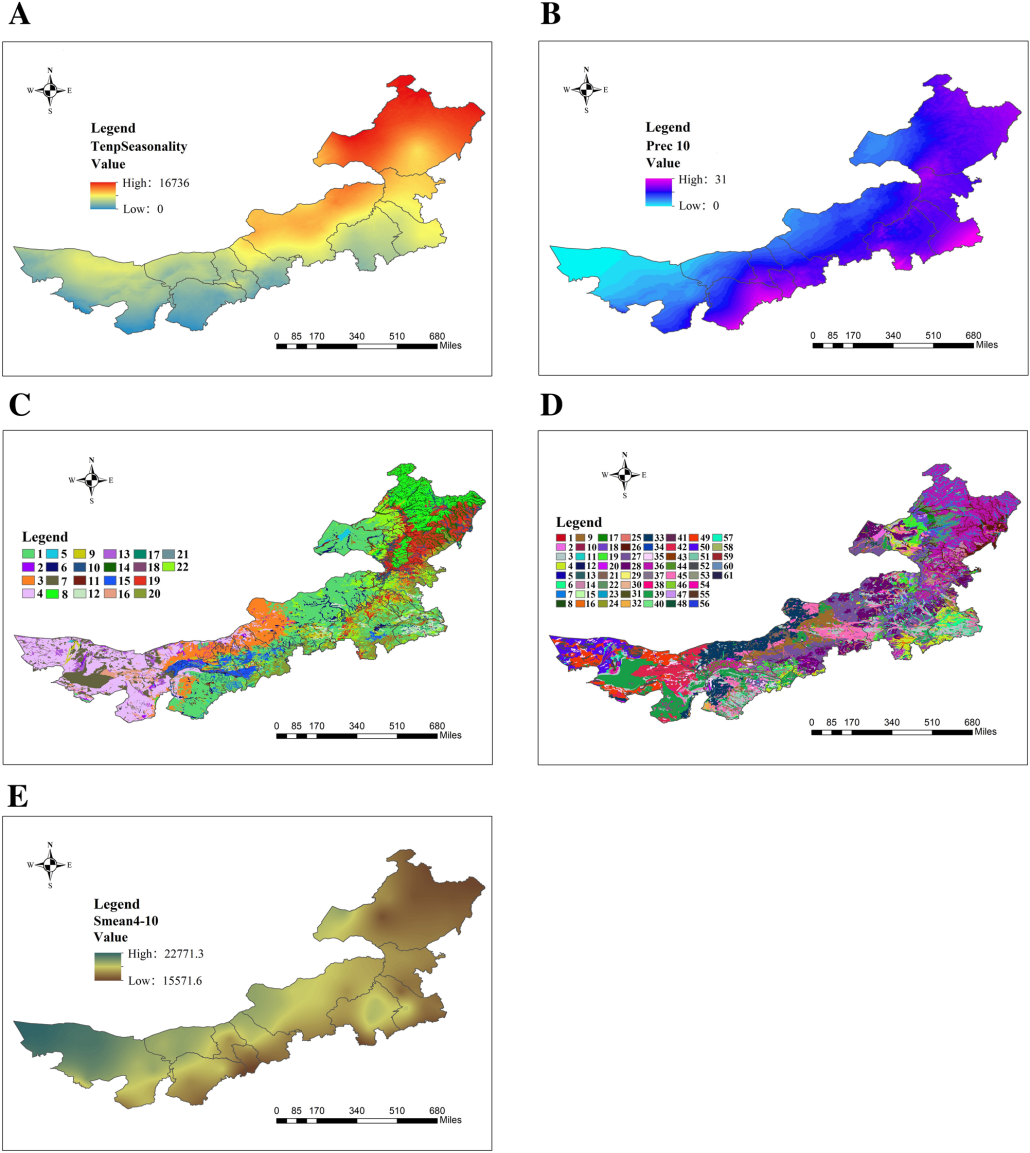
Maps of ecological environmental factors affecting *A.* *membranaceus* var. *mongholicus* distribution: tempseasonality (**A**); Prec10 (**B**); VegType (**C**) **1** temperate clump grass typical steppe; **2** temperate succulent halophyte semi-shrub desert;** 3** temperate dwarf needlegrass and dwarf semi-shrubby desert steppe; **4** temperate semi-shrub and dwarf semi-shrub desert; **5** lakes and rivers; **6** temperate grasses, miscellaneous grass, saline meadow; **7** temperate shrub desert; **8** cold-temperate and temperate mountain coniferous forest;** 9** temperate deciduous shrubland; **10** temperate grass and miscellaneous grass meadow; **11** temperate grasses, carex and miscellaneous grass swampy meadow; **12** temperate leaflet deciduous woodland; **13** temperate dwarf semi-tree desert; **14** cold-temperate and temperate swamp; **15** one harvest a year crops, cold resistant economic crops, and deciduous fruit orchard; **16** temperate steppe shrub desert; **17** temperate coniferous forests; **18** one crop annually short growing period cold-resistant crops (without fruit trees); **19** temperate deciduous broad-leaved forest; **20** one harvest a year crops and cold resistant economic crops; **21** temperate herbosa; **22** temperate grass steppe and miscellaneous steppe; SoilType (**D**) **1** Urban; **2** Inland water; **3** Cambic Arenosols; **4** Calcaric Cambisols; **5** Humic Cambisols; **6** Calcaric Fluvisols; **7** Dystric Fluvisols; **8** Umbric Gleysols; **9** Calcic Kastanozems; **10** Lithic Leptosols; **11** Eutric Regosols; **12** Haplic Solonetz; **13** Dystric Cambisols; **14** Eutric Cambisols; **15** Ferric Podzols; **16** Gleyic Cambisols; **17** Luvic Chemozems; **18** Haplic Chemozems; **19** Calcic Chemozems; **20** Rendzic Leptosols; **21** Calcic Gleysols; **22** Haplic Greyzems; **23** Mollic Gleysols; **24** Calcaric Phaeozems; **25** Gleyic Phaeozems; **26** Haplic Phaeozems; **27** Haplic Kastanozems; **28** Luvic Kastanozems; **29** Terric Histosols; **30** Gleyic Solonetz; **31** Mollic Leptosols; **32** Haplic Calcisols; **33** Luvic Calcisols; **34** Gleyic Solonchaks; **35** Calcaric Regosols; **36** Haplic Luvisols; **37** Calcic Luvisols; **38** Dystric Regosols; **39** Haplic Arenosols; **40** Stagnic Luvisols; **41** Gleyic Luvisols; **42** Haplic Gypsisols; **43** Stagnic Phaeozems; **44** Cumulic Anthrosols; **45** Calcaric Arenosols; **46** Aric Anthrosols; **47** Gleyic Chemozems; **48** Petric Calcisols; **49** Luvic Gypsisols; **50** Calcic Gypsisols; **51** Calcic Vertisols; **52** Eutric Leptosols; **53** Salic Fluvisols; **54** Mollic Solonchaks; **55** Gypsic Solonchaks; **56** Sodic Solonchaks; **57** Calcic Solonchaks; **58** Calcic Solonetz; **59** Dunes & shifting sands; **60** Fishpond; **61** Chernozems; Smean4-10 (**E**). Generated using the ArcMap version 10.5 software (ESRI Inc., California, USA. https://www.esri.com/).

As depicted in Fig. 6, 17 of the screened ecological factors primarily contributed to the growth of *A.* *membranaceus* var. *mongholicus*. The total contribution rate of TempSeasonality (40.6%), Prec10 (15.7%), VegType (14.3%), SoilType (9.2%), and Smean4_10 (9.1%) was 88.9%, thereby indicating that these five ecological factors were the key factors affecting the growth of *A.* *membranaceus* var. *mongholicus* (Fig. 7A–E).

**Figure 8:**
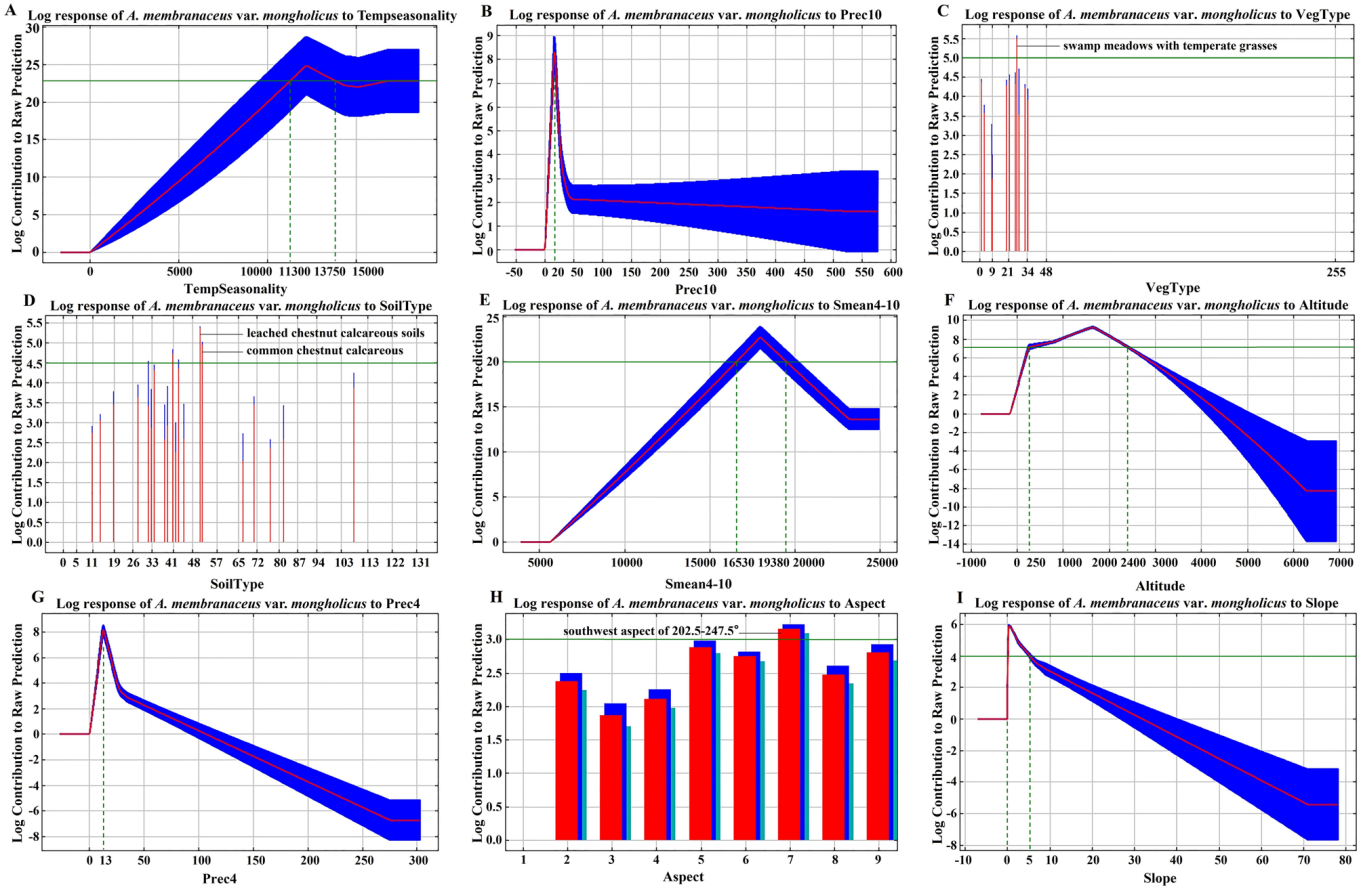

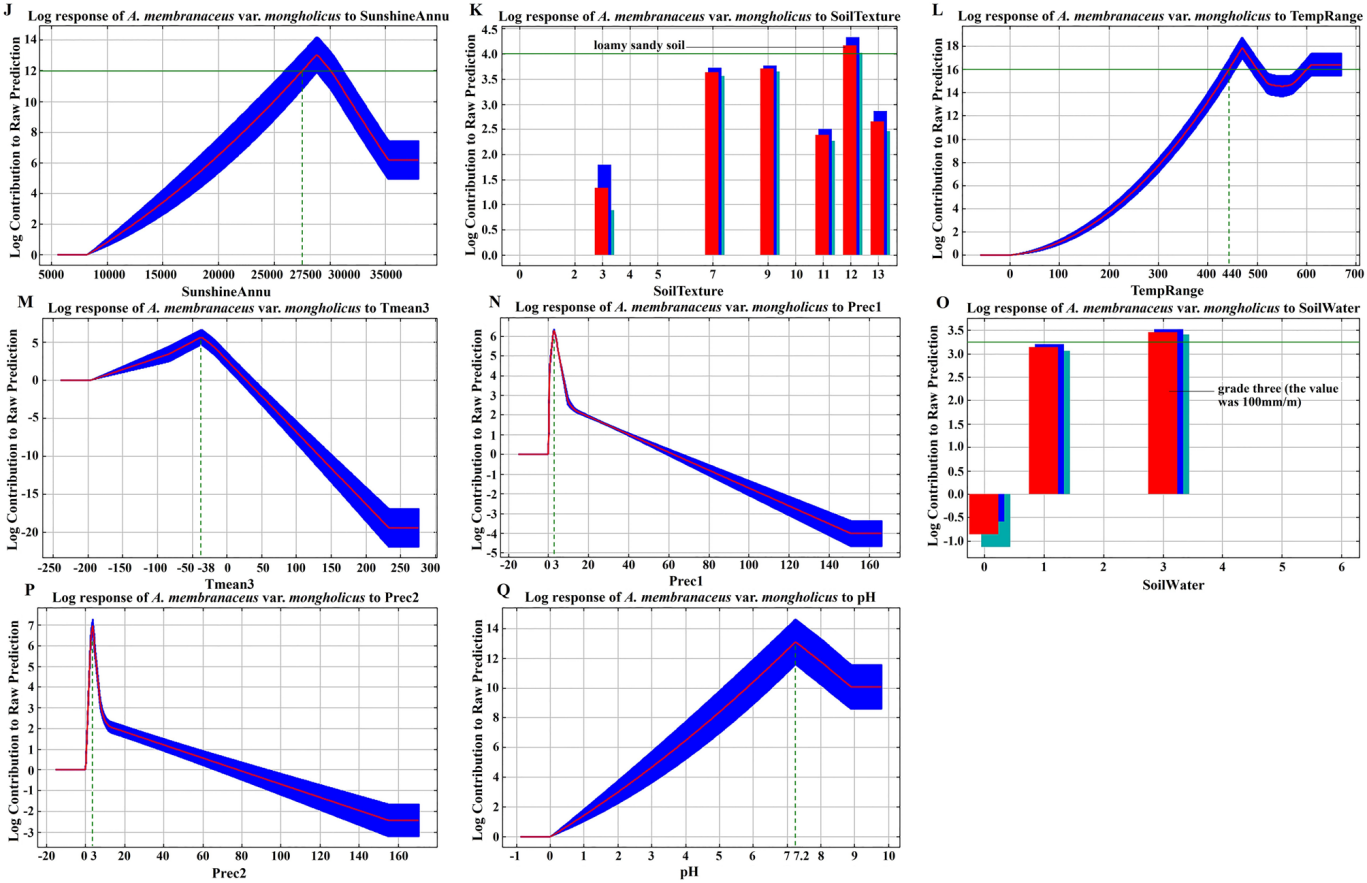
Log response of *A.* *membranaceus* var. *mongholicus* to TempSeasonality (**A**), Prec10 (**B**), VegType (**C**), SoilType (**D**), Smean4_10 (**E**), Altitude (**F**), Prec4 (**G**), Aspect (**H**), Slope (**I**), SunshineAnnu (**J**), SoilTexture (**K**), TempRange (**L**), Tmean3 (**M**), Prec1 (**N**), SoilWater (**O**), Prec2 (**P**), and PH (**Q**).

According to the response curves of each ecological factor in Fig. 8, TempSeasonality ranging from 11,300 to 13,750 was suitable for *A.* *membranaceus* var. *mongholicus* growth (Fig. 8A). The precipitation in October, April, January, and February was 20, 13, 3, and 3 mm, respectively, which was the most beneficial to *A.* *membranaceus* var. *mongholicus* growth (Fig. 8B,G,N,P); excessive rainfall did not benefit this herb. VegType conducive to the growth of *A.* *membranaceus* var. *mongholicus* was swamp meadows with temperate grasses, *Carex*, and weeds (Fig. 8C). SoilType suitable for the cultivation of *A.* *membranaceus* var. *mongholicus* was common chestnut calcareous and leached chestnut calcareous soils (Fig. 8D). Smean4_10 and SunshineAnnu ranging from 1653 to 1938 h and 2,750 to 2000 h were beneficial to *A.* *membranaceus* var. *mongholicus* growth (Fig. 8E,J). An altitude and slope of 250–2,400 m and 0°–5°, respectively, and a southwestern aspect of 202.5°‒247.5° were conducive to the growth of *A.* *membranaceus* var. *mongholicus* (Fig. 8F,I,O,H). TempAnnu of 44–50 °C and Tmean3 of -3.8 °C were most favorable for *A.* *membranaceus* var. *mongholicus* growth (Fig. 8L,M). Grade-three SoilWater (value of 100 mm/m), loamy sandy soil, and soil with a pH of 7.2 were conducive to the growth of *A.* *membranaceus* var. *mongholicus* (Fig. 8O,K,Q).

### Astragaloside IV and calycosin-7-glucoside contents in *A. membranaceus* var. *mongholicus* and their relationships with main ecological factors

Astragaloside IV and calycosin-7-glucoside contents in 63 *A.* *membranaceus* var. *mongholicus* samples are presented in Appendix 3 in Supplementary Information 1. The calycosin-7-glucoside content in all the samples reached the limit standard of *Chinese* *Pharmacopoeia*, whereas the astragaloside IV content in samples 6, 14, 15, 32, 46, 55, and 57 did not. Based on the correlation matrix for the main ecological factors with astragaloside IV and calycosin-7-glucoside, among the 17 main ecological factors, only four have a significant correlation with the content of astragaloside IV, of which Prec10, TempRange, and Tempseasonality have a significant negative correlation. Meanwhile, Prec4 has a significant positive correlation (Table 3); three ecological factors (Smean4-10, Tmean3 and pH) have a significant positive correlation with the content of calycosin-7-glucoside (Table 4). Further study on the correlation equations of astragaloside IV and calycosin-7-glucoside contents with the ecological factors were respectively established as ${y}_{1}$= 0.431 + 0.007 ${x}_{1}$−0.008 ${x}_{2}$– 0.000026 ${x}_{3}$ (${R}^{2}$= 0.542, $P$ ≤ 0.05; ${y}_{1}$= Astragaloside IV, ${x}_{1}$ = Prec4, ${x}_{2}$= Prec10, ${x}_{3}$= TempSeasonality) and ${y}_{2}$=  − 0.758 $+$ 0.049 ${x}_{1}+$ 0.000025 ${x}_{2}$ (${R}^{2}$= 0.345, $P$ ≤ 0.05; ${y}_{2}$= calycosin-7-glucoside, ${x}_{1}$= pH, ${x}_{2}$= Smean4-10). According to the analysis of the correlation matrix and correlation equations, Prec4, Prec10, and TempSeasonality are the most important ecological factors for the accumulation of astragaloside IV in *A.* *membranaceus* var. *mongholicus*. The Prec4 was beneficial to the accumulation of astragaloside IV in *A.* *membranaceus* var. *mongholicus* within a certain range, whereas TempSeasonality and Prec10 limited its accumulation. Smean4-10 and pH are the most important ecological factors for the accumulation of calycosin-7-glucoside in *A.* *membranaceus* var. *mongholicus*, which was conducive to the accumulation of this compound with a suitable soil environment and light conditions.

**Table 3:** Correlation analysis between the main ecological factors and astragaloside IV.

	Astragaloside IV (%)	VegType	PH	SoilType	SoilWater	SoilTexture	SunshineAnnu	Smean4_10	Tmean3	Prec10	Prec2	Prec4	Aspect	Slope	Altitude	TempRange	TempAnnu	TempSeasonality
Astragaloside IV (%)	1																	
VegType	0.04	1																
PH	0.10	0.12	1															
SoilType	− 0.16	0.25	− 0.07	1														
SoilWater	0.17	0.12	0.21	− 0.14	1													
SoilTexture	0.20	0.04	− 0.02	0.10	0.55**	1												
SunshineAnnu	0.04	− 0.19	− 0.09	− 0.24	− 0.08	0.19	1											
Smean4_10	0.09	− 0.07	0.16	− 0.15	− 0.07	0.13	0.79**	1										
Tmean3	0.19	0.14	0.43**	0.15	− 0.04	0.00	0.03	0.26*	1									
Prec10	− 0.42**	0.04	0.32*	0.07	− 0.01	− 0.24	− 0.27*	− 0.17	0.29*	1								
Prec2	− 0.08	0.07	0.21	0.11	0.11	− 0.16	− 0.81**	− 0.65**	0.02	0.36**	1							
Prec4	0.50**	− 0.19	0.20	− 0.15	− 0.03	− 0.01	0.05	0.01	0.01	− 0.03	0.01	1						
Aspect	− 0.06	− 0.37**	− 0.05	0.15	− 0.10	0.07	− 0.05	− 0.11	− 0.07	− 0.13	− 0.00	− 0.03	1					
Slope	− 0.10	0.06	− 0.06	− 0.15	− 0.06	− 0.04	− 0.18	− 0.17	− 0.10	0.10	0.28*	− 0.08	0.16	1				
Altitude	0.05	0.13	− 0.14	0.10	0.15	− 0.17	− 0.39**	− 0.39**	− 0.46**	− 0.31*	0.26*	0.04	0.18	0.32*	1			
TempRange	− 0.45**	− 0.11	− 0.11	0.15	− 0.02	0.02	0.08	0.08	− 0.40**	− 0.01	− 0.05	− 0.32**	0.13	− 0.19	− 0.06	1		
TempAnnu	0.18	0.03	0.26*	0.10	− 0.09	0.12	0.27*	0.38**	0.90**	0.23	− 0.20	− 0.07	− 0.12	− 0.19	− 0.67**	− 0.38**	1	
TempSeasonality	− 0.48**	− 0.14	− 0.12	− 0.06	− 0.09	0.05	0.19	0.13	− 0.48**	0.05	− 0.13	− 0.24	0.08	− 0.14	− 0.27*	0.85**	− 0.39**	1

**Significantly correlated at 0.01 level (both sides); *Significantly correlated at the 0.05 level (both sides).

**Table 4:** Correlation analysis between the main ecological factors and Calycosin-7-glucoside.

	Calycosin-7-glucoside (%)	VegType	PH	SoilType	SoilWater	SoilTexture	SunshineAnnu	Smean4_10	Tmean3	Prec10	Prec2	Prec4	Aspect	Slope	Altitude	TempRange	TempAnnu	TempSeasonality
Calycosin-7-glucoside (%)	1																	
VegType	0.24	1																
PH	0.54**	0.12	1															
SoilType	0.14	0.25	− 0.07	1														
SoilWater	0.12	0.12	0.21	− 0.14	1													
SoilTexture	0.05	0.04	− 0.02	0.10	0.55**	1												
SunshineAnnu	0.10	− 0.19	− 0.09	− 0.24	− 0.08	0.19	1											
Smean4_10	0.31*	− 0.07	0.16	− 0.15	− 0.07	0.13	0.79**	1										
Tmean3	0.33**	0.14	0.43**	0.15	− 0.04	0.00	0.03	0.26*	1									
Prec10	0.11	0.04	0.32*	0.07	− 0.01	− 0.24	− 0.27*	− 0.17	0.29*	1								
Prec2	− 0.10	0.07	0.21	0.11	0.11	− 0.16	− 0.81**	− 0.65**	0.02	0.36**	1							
Prec4	0.01	− 0.19	0.20	− 0.15	− 0.03	− 0.01	0.05	0.01	0.01	− 0.03	0.01	1						
Aspect	0.03	− 0.37**	− 0.05	0.15	− 0.10	0.07	− 0.05	− 0.11	− 0.07	− 0.13	− 0.00	− 0.03	1					
Slope	0.03	0.06	− 0.06	− 0.15	− 0.06	− 0.04	− 0.18	− 0.17	− 0.10	0.10	0.28*	− 0.08	0.16	1				
Altitude	− 0.01	0.13	− 0.14	0.10	0.15	− 0.17	− 0.39**	− 0.39**	− 0.46**	− 0.31*	0.26*	0.04	0.18	0.32*	1			
TempRange	− 0.16	− 0.11	− 0.11	0.15	− 0.02	0.02	0.08	0.08	− 0.40**	− 0.01	− 0.05	− 0.32**	0.13	− 0.19	− 0.06	1		
TempAnnu	0.19	0.03	0.26*	0.10	− 0.09	0.12	0.27*	0.38**	0.90**	0.23	− 0.20	− 0.07	− 0.12	− 0.19	− 0.67**	− 0.38**	1	
TempSeasonality	− 0.20	− 0.14	− 0.12	− 0.06	− 0.09	0.05	0.19	0.13	− 0.48**	0.05	− 0.13	− 0.24	0.08	− 0.14	− 0.27*	0.85**	− 0.39**	1

**Significantly correlated at 0.01 level (both sides); *Significantly correlated at the 0.05 level (both sides).

### Suitability regionalization of high-quality *A. membranaceus* var. *mongholicus* in Inner Mongolia

**Figure 9:**
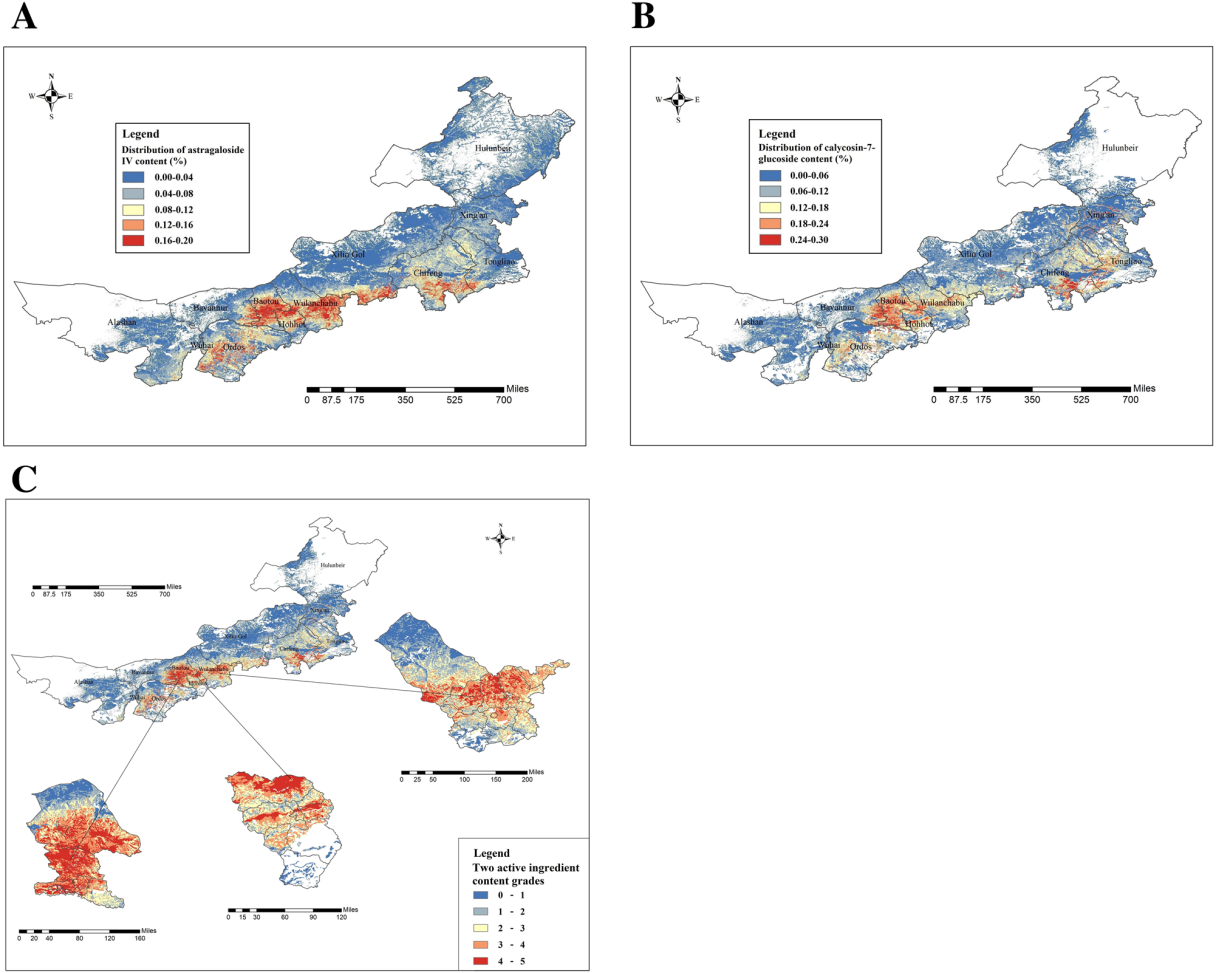
Quality suitability regionalization of *A.* *membranaceus* var. *mongholicus*: spatial distribution of astragaloside IV content (**A**); spatial distribution of calycosin-7-glucoside content (**B**); two active ingredient content grades (**C**). Generated using the ArcMap version 10.5 software (ESRI Inc., California, USA. https://www.esri.com/).

According to the spatial distribution suitability of astragaloside IV and calycosin-7-glucoside in *A.* *membranaceus* var. *mongholicus* depicted in Fig. 9A,B, areas that were the most conducive to astragaloside IV accumulation were central and western Ordos, Darhan Muminggan Joint Banner and Guyang County in Baotou City; Wuchuan County and Saihan District in Huhhot City; central and southeastern Wulanchabu, and northern Xilin Gol, Chifeng and Tongliao. Furthermore, the most favorable areas for calycosin-7-glucoside accumulation were Darhan Muminggan Joint Banner and Guyang County in Baotou City; Wuchuan County, Tumd Left Banner, Saihan and Yuquan Districts in Huhhot City; central and eastern Wulanchabu; and southern Chifeng City. Table 5 presents the level of suitability for astragaloside IV and calycosin-7-glucoside distribution at various administrative levels (e.g., banner, district, county, and city).

**Table 5:** Administrative areas under various suitability level of astragaloside IV and calycosin-7-glucoside distribution.

Leagues or cities	Optimum area		Suitable area		Secondarily suitable and unsuitable areas	
	Astragalosude IV content in the range of 0.12–0.20%	Calycosin-7-glucoside content in the range of 0.18–0.30%	Astragalosude IV content in the range of 0.04–0.12%	Calycosin-7-glucoside content in the range of 0.06–0.18%	Astragalosude IV content < 0.04%	Calycosin-7-glucoside content < 0.06%
Alashan League	Alashan Left Banner	Alashan Left Banner	Alashan Left Banner, Alashan Right Banner	Alashan Left Banner, Alashan Right Banner	Ejin Banner, Alashan Left Banner, and Alashan Right Banner	Ejin Banner, Alashan Left Banner, and Alashan Right Banner
Wuhai City	Hainan District, Haibowan District	Hainan District	Hainan District, Haibowan District, Wuda District	Hainan District, Haibowan District, Wuda District	Haibowan District, Wuda District	Hainan District, Haibowan District, Wuda District
Bayannur League	Urat Middle Banner and Urat Front Banner	Urat Middle Banner and Urat Front Banner	Urat Back Banner, Urat Middle Banner, Urat Front Banner, Dengkou County, and Wuyuan County	Urat Back Banner, Urat Middle Banner, Urat Front Banner, Dengkou County, and Wuyuan County	Urat Back Banner, Urat Middle Banner, Dengkou County, Hanggin Back Banner, Linhe Distrsct, and Wuyuan County	Urat Back Banner, Urat Middle Banner, Dengkou County, Hanggin Back Banner, Linhe Distrsct, and Wuyuan County
Ordos City	Hanggin Banner, Otog Banner, Otog Front Banner, Uxin Banner, Ejin Horo Banner, Dongsheng District, Dalad Banner	Hanggin Banner, Otog Banner, Uxin Banner, Ejin Horo Banner, Dalad Banner, Jungar Banner	Hanggin Banner, Otog Banner, Otog Front Banner, Uxin Banner, Ejin Horo Banner, Dongsheng District, Dalad Banner, and Jungar Banner	Hanggin Banner, Otog Banner, Otog Front Banner, Ejin Horo Banner, Dongsheng District, Dalad Banner, Jungar Banner	Hanggin Banner, Otog Front Banner, Uxin Banner, Ejin Horo Banner, and Jungar Banner	Hanggin Banner, Otog Banner, Otog Front Banner, Uxin Banner, Ejin Horo Banner, Dongsheng District, Dalad Banner, Jungar Banner
Baotou City	Darhan Muminggan Joint Banner, Bayan Obo Mining District, Guyang County, Shiguai District, and Tumd Right Banner	Darhan Muminggan Joint Banner, Bayan Obo Mining District, Guyang County, Shiguai District, and Tumd Right Banner	Darhan Muminggan Joint Banner and Tumd Right Banner	Darhan Muminggan Joint Banner, Guyang County, Hondlon District, and Tumd Right Banner	Hondlon District and Hondlon District	Darhan Muminggan Joint Banner, Guyang County, Hondlon District, and Tumd Right Banner
Huhhot City	Wuchuan County, Tumd Left Banner, Xicheng District, Saihan District, Huiming District, Yuquan District, Togtoh County, and Horinger	Wuchuan County, Tumd Left Banner, Saihan District, Yuquan District, and Togtoh County	Tumd Left Banner, Xicheng District, Huiming District, Togtoh County, and Horinger	Wuchuan County, Tumd Left Banner, Xicheng District, Saihan District, Huiming District, Yuquan District, Togtoh County, and Horinger	Tumd Left Banner, Togtoh County, Horinger, and Qingshuihe County	Wuchuan County, Tumd Left Banner, Xicheng District, Huiming District, Horinger, and Qingshuihe County
Wulanchabu City	Siziwang Banner, Chahar Right Wing Middle Banner, Chahar Right Wing Back Banner, Shangdu County, Huade County, Zhuozi County, Chahar Right Wing Front Banner, Xinghe County, and Feng Chin	Siziwang Banner, Chahar Right Wing Middle Banner, Chahar Right Wing Back Banner, Shangdu County, Huade County, and Zhuozi County	Siziwang Banner, Chahar Right Wing Middle Banner, Huade County, Zhuozi County, Chahar Right Wing Front Banner, Xinghe County, Langcheng County, and Feng Chin	Siziwang Banner, Chahar Right Wing Middle Banner, Chahar Right Wing Back Banner, Shangdu County, Huade County, Zhuozi County, Chahar Right Wing Front Banner, Xinghe County, and Feng Chin	Siziwang Banner and Langcheng County	Siziwang Banner, Chahar Right Wing Middle Banner, Huade County, Zhuozi County, Chahar Right Wing Front Banner, Xinghe County, Langcheng County, and Feng Chin
Xilin Gol	Sonid Right Banner, Xianghuang Banner, Zhengxiangbai Banner, Taibus Banner, Zhenglan Banner, Duolun Banner, and Xilinhot	Sonid Right Banner, Zhengxiangbai Banner, Taibus Banner, Zhenglan Banner, Duolun Banner, Abag Banner, Xilinhot, West Ujimqin Banner, and East Ujimqin Banne	Sonid Right Banner, Xianghuang Banner, Zhengxiangbai Banner, Zhenglan Banner, Duolun Banner, Abag Banner, Xilinhot, West Ujimqin Banner, and East Ujimqin Banner	Erenhot, Sonid Right Banner, Sonid Left Banner, Zhengxiangbai Banner, Xianghuang Banner, Taibus Banner, Zhenglan Banner, Duolun Banner, Xilinhot, Abag Banner, West Ujimqin Banner, and East Ujimqin Banne	Erenhot, Sonid Right Banner, Sonid Left Banner, Zhengxiangbai Banner, Zhenglan Banner, Duolun Banner, Abag Banner, Xilinhot, West Ujimqin Banner, and East Ujimqin Banner	Erenhot, Sonid Right Banner, Sonid Left Banner, Zhengxiangbai Banner, Taibus Banner, Zhenglan Banner, Duolun Banner, Xilinhot, Abag Banner, West Ujimqin Banner, and East Ujimqin Banne
Chifeng City	Hexigten Banner, Ongniud Banner, Songshan District, Yuanbaoshan District, Hongshan District, Yuanbaoshan District, Harqin Banner, and Aohan Banner	Ar Horqin Banner, Bairin Left Banner, Bairin Right Banner, Hexigten Banner, Ongniud Banner, Songshan District, Yuanbaoshan District, Harqin Banner, and Aohan Banner	Ar Horqin Banner, Bairin Left Banner, Bairin Right Banner, Linxi County, Hexigten Banner, Ongniud Banner, Songshan District, Harqin Banner, Ningcheng County, and Aohan Banner	Ar Horqin Banner, Bairin Left Banner, Bairin Right Banner, Linxi County, Hexigten Banner, Ongniud Banner, Songshan District, Harqin Banner, Ningcheng County, and Aohan Banner	Ar Horqin Banner, Bairin Right Banner, Hexigten Banner, Ongniud Banner, Harqin Banner, and Ningcheng County	Ar Horqin Banner, Bairin Right Banner, Hexigten Banner, Ongniud Banner, Harqin Banner, Ningcheng County, and Aohan Banner
Tongliao City	Naiman Banner, Kulun Banner, and Jarud Banner	Naiman Banner, Kulun Banner, Kailu County, Horqin Left Wing Middle Banner, Jarud Banner, and Holingola	Naiman Banner, Kulun Banner, Kailu County, Horqin Left Wing Middle Banner, Jarud Banner, and Holingola	Naiman Banner, Kulun Banner, Kailu County, Horqin District, Horqin Left Wing Middle Banner, Jarud Banner, and Holingola	Naiman Banner, Kulun Banner, Horqin Left Wing Middle Banner, Horqin District, Horqin District, and Horqin Left Wing Back Banner, Horqin District	Naiman Banner, Kulun Banner, Horqin District, Horqin Left Wing Back Banner, Horqin Left Wing Middle Banner, and Jarud Banner
Xing'an League	–	Arxan, Jalaid Banner, Horqin Right Front Banner, Ulanhot, Tuquan County, Horqin Right Middle Banner	Jalaid Banner, Horqin Right Front Banner, Ulanhot, Tuquan County, Horqin Right Middle Banner	Jalaid Banner, Horqin Right Front Banner, Ulanhot, Tuquan County, Horqin Right Middle Banner	Arxan, Jalaid Banner, Horqin Right Front Banner, Horqin Right Middle Banner	Arxan, Jalaid Banner, Horqin Right Front Banner, Ulanhot, Tuquan County, Horqin Right Middle Banner
Hulunbeir City	Oroqen Autonomous Banner	–	Oroqen Autonomous Banner, Daur Autonomous Banner, Arun Banner, Zhalantun, Yakeshi, Genhe, and Erguna	Zhalantun, Erguna, Chen Barag Banner, Ewenki Autonomous Banner, and Xin Barag Left Banner	Oroqen Autonomous Banner, Daur Autonomous Banner, Arun Banner, Zhalantun, Yakeshi, Genhe, Erguna, Chen Barag Banner, Hailar District, Ewenki Autonomous Banner, Xin Barag Left Banner, Manzhouli, and Xin Barag Right Banner	Oroqen Autonomous Banner, Daur Autonomous Banner, Arun Banner, Yakeshi, Zhalantun, Genhe, Erguna, Chen Barag Banner, Hailar District, Ewenki Autonomous Banner, Xin Barag Left Banner, Manzhouli, and Xin Barag Right Banner

We estimated the positive and negative effects of various ecological factors in Inner Mongolia on the astragaloside IV and calycosin-7-glucoside content to analyze the spatial distribution of the two index components. We consequently found that Guyang County in Baotou City; Wuchuan County in Hohhot City; and central Wulanchabu City (Chahar Right Middle Banners, Chahar Right Back Banners, and Shangdu County) were not only suitable for the growth of *A.* *membranaceus* var. *mongholicus,* but also conducive to the accumulation of the abovementioned index components (Fig. 9C). The quality of *A.* *membranaceus* var. *mongholicus* produced in the aforementioned areas was the highest; this finding can be used as a reference for the cultivation of high-quality *A.* *membranaceus* var. *mongholicus* throughout Inner Mongolia. In addition to traditional production areas, such as Guyang County in Baotou City and Wuchuan County in Hohhot City, Chahar Right Middle Banner, Chahar Right Back Banner, and Shangdu County in Wulanchabu City were potentially the most suitable areas for producing high-quality *A.* *membranaceus* var*.* *mongholicus.*

## Discussion

Considerable effort was made to expand the collection and increase the number of *A.* *membranaceus* var. *mongholicus* samples, which reached > 90% of the total known growth area in this study. Owing to limiting factors, such as climatic and environmental conditions, topography, and time constraints during sampling, some locations where *A.* *membranaceus* var. *mongholicus* grows may have been overlooked and not sampled. This shortcoming is believed to have slightly affected our results; hence, future studies should more comprehensively identify areas where this species grows and incorporate such samples into analyses.

In this study of ecological suitability regionalization for *A.* *membranaceus* var. *mongholicus*, the response curves of the main ecological factors reflect the most suitable environment for the growth of this species. According to the biological understanding of *A.* *membranaceus* var. *mongholicus*, the climatic factors such as temperature (Tempseasonality, TempRange, and Tmean3), precipitation (Prec10, Prec4, Prec1, and Prec2) and sunshine duration (Smean4-10, SunshineAnnu); soil (soil pH, SoilWater, SoilTexture, and SoilType) and topographic (slope, aspect, and altitude) factors; and the biological factor (VegType) analyzed in this study, explain the niche of this species based on the accurate prediction results of the Maxent model. However, for medicinal plants, it is worth noting that the environment suitable for growth does not necessarily accumulate more active components. Many experiments of environmental stress related to medicinal plants have shown that harsh environments may induce them to accumulate more active components^28–29^. This phenomenon explains why the response curves of the main ecological factors involved in this study are favorable to *A.* *membranaceus* var. *mongholicus* growth limited to a certain range.

Correlations between the index components in *A.* *membranaceus* var. *mongholicus* and ecological factors showed that the accumulation of astragaloside IV was primarily affected by climatic factors, including temperature and precipitation (TempSeasonality, Prec4, and Prec10), whereas the accumulation of calycosin-7-glucoside was mainly affected by soil factors and sunshine duration (soil pH and Smean4-10). Compared with the accumulated calycosin-7-glucoside, that of astragaloside IV in *A.* *membranaceus* var. *mongholicus* was more susceptible to the influence of the ecological environment, and different ecological factors had positive and negative effects on its growth. Therefore, as the main bioactive saponin in *A.* *membranaceus* var. *mongholicus* and the saponin extract commonly used in the market^30,31^, astragaloside IV can be obtained in large quantities by artificially controlling the ecological environment of the production area to cultivate high-quality herbs rich in this extract.

In this study of suitability regionalization of high-quality *A.* *membranaceus* var. *mongholicus*, the positive and negative effects of various ecological factors on the accumulation of astragaloside IV and calycosin-7-glucoside were comprehensively analyzed. The most suitable areas for the growth of high-quality *A.* *membranaceus* var. *mongholicus* were found to be Guyang County in Baotou City; Wuchuan County in Hohhot City; and central Wulanchabu City (Chahar Right Middle Banner, Chahar Right Back Banner, and Shangdu County). These regions located in the Yinshan Mountains have an important geographical location (the geographic boundary of east–west mountains in northern China) and unique ecological environment (ecological barriers in central Inner Mongolia). Therefore, the areas are influenced by topography and climate and have distinct seasons with little precipitation but significant differences in temperature, which are conducive to the accumulation of secondary metabolites in *A.* *membranaceus* var. *mongholicus*. The requirements of this species in terms of soil are not specific, but soil texture and the thickness of the soil layer affect the yield and quality of this herb^32^. The prediction results of the study further show that the most suitable distribution areas were mostly located in well-drained, high terrain areas.

At present, an increasing number of Chinese patent medicines, food, health products, and cosmetics are produced using *A.* *membranaceus* var. *mongholicus* as a raw material^33,34^. For example, Shenqi granules from Sun Flower, Huangqi tablets, Huangqi Danggui Ajiao from Tongrentang Chinese Medicine, and Chunjuan Astragalus Cream. Although several high-quality products are produced by reputable companies, healthcare professionals, patients, and consumers are concerned about the questionable botanical ingredients present in various consumer products^35^. By understanding the internal and external links between production, processing, and trade networks, the quality of medicinal plant products may be more clearly discerned in different markets^36^. The value addition of medicinal plants can be introduced at different stages of their production. As the first step of its value chain, the cultivation technology of *A.* *membranaceus* var. *mongholicus* is essential for increasing its value^37^. In our study on the ecological and quality suitability regionalization of *A.* *membranaceus* var. *mongholicus*, the quality of this medicinal plant is closely related to site selection for cultivation. Therefore, the realization of value in the process of transforming raw materials from medicinal plants into high-value products not only depends on actual production, but scientific guidance is also an indispensable step.

Notably, this study involved the application of theoretical knowledge from various disciplines, including meteorology, pharmacology, and computer science. Interdisciplinary research to advance the traditional Chinese medicine industry is expected to improve the current understanding on scientific research, through which a variety of scientific research methods may emerge^38^. Combining different methods is likely to be conducive to the industrialization of traditional Chinese medicine. However, this field is still in its infancy, and limited research technology and understanding of the influence of different, seemingly unrelated, factors substantially limit the progress of scientific research^39^. Therefore, although new theories and technologies explored during interdisciplinary research may face challenges, multidisciplinary research can reveal meaningful scientific truths about traditional Chinese medicine.

This study predicted the ecological suitability and distribution areas for cultivating high-quality *A.* *membranaceus* var. *mongholicus* based on environmental factors in Inner Mongolia. However, human disturbances also have important effects on the quality of *A.* *membranaceus* var. *mongholicus* during cultivation^40^. In fact, in terms of both environmental and anthropogenic factors, no single factor is expected to completely control the quality of raw medicinal plant materials. Rather, combinations of these factors likely offer the best control of medicinal material quality^41^. Therefore, in the cultivation and production of *A.* *membranaceus* var. *mongholicus*, in addition to considering environmental factors (e.g., climate, soil, and topography), anthropogenic factors (e.g., soil fertility and irrigation) also need to be considered to achieve high-yield and -quality crops.

## Conclusions

The suitability regionalization of high-quality *A.* *membranaceus* var. *mongholicus* in Inner Mongolia showed that Guyang County in Baotou City; Wuchuan County in Hohhot City; and central Wulanchabu City (Chahar Right Middle Banner, Chahar Right Back Banner, and Shangdu County) and its surroundings were favorable for the cultivation of *A.* *membranaceus* var. *mongholicus* and accumulation of its active compounds. The quality of *A.* *membranaceus* var. *mongholicus* produced in these areas was the best, and Chahar Right Middle Banner, Chahar Right Back Banner, and Shangdu County in Wulanchabu City were potentially the most suitable areas for producing high-quality crops. This study can be used to inform site selection for cultivating this species to promote the development of a standardized, good agriculture practice production base in Inner Mongolia as well as to nurture and protect *A.* *membranaceus* var. *mongholicus* resources.

## Methods

### Materials

BioSim2 version 2 (Information Technology Department of Norwich University, Las Vegas, NE, USA), Maxent version 3.3.3 (AT&T Labs–Research, Florham Park, NJ, USA; https://www.cs.princeton.edu/~schapire/maxent/), and ArcGIS version 10.5 (ESRI Inc., California, USA; https://www.esri.com/) software were respectively used for screening the environmental variables, predicting the *A.* *membranaceus* var. *mongholicus* habitat, and mapping the suitable distribution area of *A.* *membranaceus* var. *mongholicus* in Inner Mongolia. The geographic coordinate information of *A.* *membranaceus* var. *mongholicus* samples was obtained by Handheld GPS (Jarmin Rino530HCx, Nanjing Tiandi Precision Drawing Instrument Equipment Co. Ltd., Nanjing).

The extraction of active components (Astragaloside IV and calycosin-7-glucoside) from *A.* *membranaceus* var. *mongholicus* samples was carried out by experimental equipment, namely pulverizer (FLBP-200, Zhejiang Yili Industry and Trade Co., Ltd., Zhejiang), electronic analytical balance (ME204, Mettle Toledo, Shanghai), temperature-controlled electric heating jacket (KDM, Heze Jingke Instrument Co., Ltd., Heze), rotary evaporator (IKA RV 10, Shanghai Hanpei Electromechanical Equipment Co. Ltd., Shanghai), ultrasonic cleaner (KQ-500DE, Sonxi Ultrasonic, Kunshan), the Soxhlet extractor, reflux extractor, volumetric bottle, and other glass instruments produced by Shuniu Glass Instrument Co., Ltd. (Sichuan). High-performance liquid chromatography-ultraviolet (Ultimate3000, Thermo Fisher Scientific, Waltham, MA, USA), evaporative light scattering detector (ELSD 2000ES, Alltech (Shanghai in China) Co., Ltd.), air generator (XWK-III, Tianjin Jinmin Analytical Instrument Manufacturing Co., Ltd., Tianjin), and Astragaloside IV (CAS: 148321) and calycosin-7-glucoside (CAS: 150326) reference substances of > 95% purity purchased from Chengdu Pufeide Biotech Co., Ltd (Chengdu) were used for the quantitative analyses of the samples. A laboratory water purifier (XGB-40-B, Shenyang Xinjie Technology Co., Ltd., Shenyang), chromatographic methanol, n-butanol, and phosphoric acid were purchased from Tianjin Fuchen Chemical Reagent Factory, chromatographic acetonitrile was purchased from Tianjin Comeo Chemical Reagent Co., Ltd., and ammonia water was purchased from Tianjin Fengchuan Chemical Reagent Technology Co., Ltd (Tianjin) for the entire High-performance liquid chromatography-ultraviolet analysis.

### Acquisition of ecological factor data

In this study, we considered the influence of 74 ecological factors, including climate, soil, topography, vegetation type, and meteorological factors, on the distribution of *A.* *membranaceus* var. *mongholicus*. Climatic data included 59 ecological factors, e.g., monthly mean precipitation (mm), temperature (°C × 10), and sunshine duration (h × 10) from January to December (mm), mean precipitation (mm), temperature (Tmean4-10, °C × 10), and sunshine duration (Smean4-10, h × 10) in the growing season, mean annual sunshine duration (SunshineAnnu, h × 10), and 19 comprehensive climatic factors. These data were based on spatial interpolation of meteorological observation data from 752 surface and automatic meteorological stations in China, collected from 1951 to 2000, with a resolution of 1 km.

The soil data comprised eight ecological factors, which were determined according to a 1:100,000 soil map of the People’s Republic of China (compiled in 1995) provided by the Second National Land Survey. These factors were soil pH, cation exchange capacity (cmol kg^−1^), sand content (SoilSand, %), clay content (%), soil type (SoilType from FAO-90), soil available water content level (SoilWater), soil texture (SoilTexture, USDA), and organic carbon content (SoilCarbon, %).

Topographic data included three ecological factors, namely altitude (m), slope (°), and aspect with a resolution of 1 km. Vegetation type data included an ecological factor based on vegetation subtype data from a vegetation map of the People’s Republic of China (1:100,000) published by the Institute of Botany, Chinese Academy of Sciences. The comprehensive meteorological data comprised three ecological factors, namely the warmth and coldness indexes (°C) derived from Kira’s thermal index and the humidity index (mm∙°C^−1^) derived from Xu’s modified version of Kira’s humidity index^42,43^.

The abovementioned data of ecological factors for studying the ecological suitability and quality regionalization of *A.* *membranaceus* var. *mongholicus* were taken from the “Traditional Chinese Medicine Resources Spatial Information Grid Database” (https://www.tcm-resources.com/) provided by the National Resource Center for Chinese Materia Medica of the China Academy of Chinese Medical Sciences (Beijing, China). The relevant information for each ecological factor, including the category, name and type, is presented in Appendix 1 in Supplementary Information 1.

### Collection of *A. membranaceus* var. *mongholicus* samples

**Figure 10:**
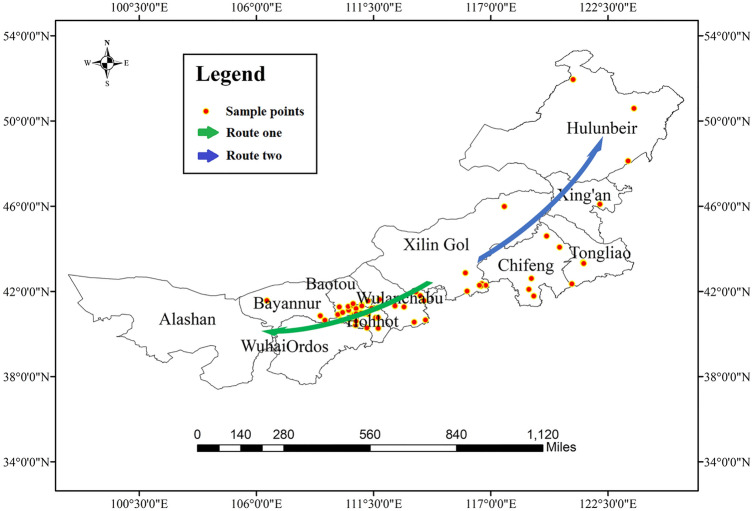
Survey routes of *A.* *membranaceus* var. *mongholicus* and geographical location of its sampling points. Generated using the ArcMap version 10.5 software (ESRI Inc., California, USA. https://www.esri.com/).

The cultivation area of *A.* *membranaceus* var. *mongholicus* was approximately 6,666.67 ha (66.67 km^2^) in Inner Mongolia in 2016 and mainly covered Urad Front Banner, Guyang County, Tumd Right Banner, Wuchuan County, and Harqin Banner^44^. Based on a full understanding of the regional characteristics of Inner Mongolia, we adopted traditional route and quadrat surveys, taking the village or *gacha* (level with administrative village) as the smallest sampling unit, to conduct a field survey of *A.* *membranaceus* var. *mongholicus* in the eastern, central, and western regions of Inner Mongolia in 2016. To ensure the uniformity and representativeness of the sample data, we sampled different production areas with significant differences in ecological factors, such as terrain, soil, and vegetation type, according to these two routes. Three to five sampling points were set in each sampling area with a distance of 1 km, and a total of 63 samples of *A.* *membranaceus* var. *mongholicus* were collected in Inner Mongolia. The geographic coordinates of the sampling points were recorded using the handheld GPS. To diminish the influence of different harvesting periods or growth years on the contents of active components in *A.* *membranaceus* var. *mongholicus*, the samples were biennial medicinal materials and were collected in October 2016. Figure 10 shows the survey route and location of the sampling points. The geographical coordinates (latitude and longitude data) of each sampling point are listed in Appendix 4 in Supplementary Information 1.

### Preliminary screening of ecological factors

To diminish the influence of high correlations between ecological factors at the time of MaxEnt modeling, we used Biosim2 to calculate the correlation coefficients between all the ecological factor values extracted from the longitude and latitude of 63 sampling points. According to the correlation coefficient tree diagram calculated using Biosim2, we excluded any ecological factor that had a low correlation with *A.* *membranaceus* var. *mongholicus* growth, based on their potential biological relevance to this species, for each set of highly correlated ecological factors with correlation coefficients > 0.8^45^. We loaded the retained ecological factors into Biosim2 again and repeated the above-mentioned operation until the correlation coefficient between all the retained ecological factors was ≤ 0.8. The ecological factors finally screened are depicted in Fig. 3.

### Calculation and accuracy testing of MaxEnt model

The point locality data of *A.* *membranaceus* var. *mongholicus* and the retained ecological factor data were imported into the MaxEnt model for the calculation. The model parameters were set as follows: The model was run 10 times, the maximum number of iterations was 1,000,000, the convergence threshold was 0.0005, the random test percentage was set to 10, namely, 90% of the point locality data were randomly selected as training data, and the remaining 10% of data points were the test data. Cross validation (the data set was divided into ten parts, of which 9 were used as training data and 1 as test data in turn for the experiment) was used as the replicated run type, and the max number of background points and the remaining parameters were set as default.

In this study, the receiver operating characteristic curve analysis of the distribution of *A.* *membranaceus* var. *mongholicus* in the model was used to evaluate the accuracy of MaxEnt. The area under the receiver operating characteristic (AUC) was not affected by the threshold, and its value ranged from 0 to 1. The larger the value, the higher was the accuracy of the model. When the AUC was in the range 0.5–0.8, the accuracy of the prediction made by the model was inferior; however, the prediction accuracy was reasonable when the AUC was in the range 0.8–0.9. Finally, when the AUC was greater than 0.9, the model produced reliable and accurate prediction results, and the potential distribution of the species could be accurately predicted^46,47^. The results of 10 training and test sample data repeatedly calculated using MaxEnt are presented in Table 1, and the mean AUC and standard deviation value of the test samples are depicted in Fig. 4. The average growth suitability image of *A.* *membranaceus* var. *mongholicus* obtained through the model was used as the probability layer file of potential *A.* *membranaceus* var. *mongholicus* distribution for studying the ecological suitability regionalization of *A.* *membranaceus* var. *mongholicus*.

### Ecological suitability regionalization of *A. membranaceus* var.* mongholicus* in Inner Mongolia

The point locality data of *A.* *membranaceus* var. *mongholicus* and its average growth suitability image (distribution probability layer of this species) were simultaneously loaded into ArcGIS. The distribution data of the *A.* *membranaceus* var. *mongholicus* sampling points were used to extract the ecological suitability values in the distribution probability layer, which was rasterized according to the maximum and minimum values of the suitability value. This was done to remove the data outside the range of the sampling points and obtain the region suitable for the cultivation of *A.* *membranaceus* var. *mongholicus*. The natural breaks method in ArcGIS was used to divide the ecological suitability distribution area of the species into four levels: unsuitable (0.00–0.02), secondarily suitable (0.02–0.18), suitable (0.18–0.42), and optimum (0.42–0.90); we used an appropriate color ramp in ArcGIS to indicate the aforementioned levels. Finally, a legend, north arrow, and scale bar were added to complete the map of the ecological suitability of *A.* *membranaceus* var. *mongholicus* at the city level in Inner Mongolia (Fig. 5). To accurately obtain the potential distribution area of *A.* *membranaceus* var. *mongholicus*, based on the ecological suitability of this species in Inner Mongolia, we added county-level administrative data to ArcGIS. Next, we extracted the distribution probability layer of this species by mask to obtain a map of the ecological suitability of *A.* *membranaceus* var. *mongholicus* at the county level. Subsequently, the areas of suitable habitat in the Leagues or Cities in Inner Mongolia were statistically analyzed (Table 2).

### Main ecological factors affecting *A. membranaceus* var.* mongholicus* growth

The ecological factors screened by Biosim2 were inputted as environmental variables into the Maxent model for model calculation, and the contribution rate of each factor to the growth of *A.* *membranaceus* var. *mongholicus* was determined. To determine the first estimate, in each iteration of the training algorithm the increase in regularized gain was added to the contribution of the corresponding variable, or subtracted from it if the change to the absolute value of *λ* was negative. For the second estimate, for each environmental variable, the values of that variable on training presence and background data were randomly permuted. Finally, the model was reevaluated on the permuted data and the contribution of each factor was obtained (Fig. 6). Ecological factors with a contribution rate of > 0% were selected as the main factors to analyze the response curves (Fig. 8A–Q). Those contributing to the growth of the species were used as the main ecological factors for studying the suitability regionalization of high-quality *A.* *membranaceus* var. *mongholicus* in Inner Mongolia.

### Content of index components and relationships with main ecological factors

Saponins and flavonoids are the primary active components of Radix Astragali, and they are valuable indicators for evaluating the quality of Radix Astragali in the Chinese, British, and European pharmacopoeia^3,48,49^. The contents of the saponin astragaloside IV and flavonoid calycosin-7-glucoside in 63 Radix Astragali samples were determined via high performance liquid chromatography according to the *Chinese* *Pharmacopoeia* (2015 Edition) (Appendix 3 in Supplementary Information 1). In addition, we used SPSS17.0 statistical analysis software to analyze differences in astragaloside IV and calycosin-7-glucoside content in *A.* *membranaceus* var. *mongholicus* from different production areas in Inner Mongolia. The relationships between astragaloside IV, calycosin-7-glucoside, and the main ecological factors were determined using the correlation matrix (Tables 3, 4). The relationship equations between these index components and the main ecological factors were obtained by stepwise linear regression analysis.

### Suitability regionalization of high-quality *A. membranaceus* var.* mongholicus* in Inner Mongolia

The relationship equations were respectively inputted into ArcGIS’s grid calculator to obtain the quantitative distribution layers of astragaloside IV and calycosin-7-glucoside in *A.* *membranaceus* var. *mongholicus*. Using the spatial calculation function of ArcGIS, the two abovementioned layers were overlain on the ecological suitability distribution layer of *A.* *membranaceus* var. *mongholicus*, and the spatial suitability distribution regions of astragaloside IV and calycosin-7-glucoside in *A.* *membranaceus* var. *mongholicus* in Inner Mongolia were finally obtained. According to the content limits of these index components in 63 *A.* *membranaceus* var. *mongholicus* samples, the spatial suitability distribution regions of the index components were divided into five grades in ArcGIS, represented by a color ramp from blue to red. A map of the spatial distribution of astragaloside IV and calycosin-7-glucoside in *A.* *membranaceus* var. *mongholicus* in the study area was plotted in ArcGIS (Fig. 9A,B). To determine the regions in Inner Mongolia that were suitable for cultivating high-quality *A.* *membranaceus* var. *mongholicus*, we overlaid the spatial distribution layer of the two active ingredients and the administrative distribution data at the county level to find out where these contents are both maximized (Fig. 9C). The administrative areas under various suitability levels of astragaloside IV and calycosin-7-glucoside distribution are presented in the Table 5.

## Supplementary information

Supplementary file1 (DOC 293 kb)
