# Supplementary material for: Ecological niche modeling of Astragalusmembranaceus var. mongholicus medicinal plants in Inner Mongolia, China
Source: Sci Rep. 2020 Jul 27;10:12482. doi: 10.1038/s41598-020-69391-3 (PMC7385632; doi:10.1038/s41598-020-69391-3)
Supplement: Supplementary file 1 — Supplementary file1 (DOC 293 kb) [file 41598_2020_69391_MOESM1_ESM.doc]

Ecological niche modeling of *Astragalus membranaceus* var. *mongholicus* medicinal plants in Inner Mongolia, China

Min Yang^1,†^, Ziyan Li^1,†^, Lanbo Liu^2^, Agula Bo^6^, Chunhong Zhang^1,3*^ & Minhui Li1,4,5,6[[1]](#footnote-2)*

*^1^Department of Pharmacy, Baotou Medical College, Baotou 014060, China*

*^2^Baotou Meteorological Bureau of Inner Mongolia Autonomous region, Baotou* *014030, China*

*^3^Inner Mongolia key laboratory of Chinese Medicinal Materials Resource, Baotou Medical College, Baotou 014060, China*

*^4^Pharmaceutical laboratory, Inner Mongolia Institute of Traditional Chinese Medicine, Hohhot 010020, China*

*^5^Guangxi Key Laboratory of Medicinal Resources Protection and Genetic Improvement, Guangxi Botanical Garden of Medicinal Plants, Nanning 530023, China*

*^6^Inner Mongolia Key Laboratory of Characteristic Geoherbs Resources Protection and Utilization, Baotou Medical College, Baotou 014060, China*

^†^ These authors contributed to the paper equally.

Supplementary Material

Appendix 1. Details of 74 ecological factor data.

| **NO.** | **Category** | **Spatial resolution or origin** | **Name** | **Abbreviation** | **Type** | **Implication** | **Excluded or included after Biosim2 screening** |
| --- | --- | --- | --- | --- | --- | --- | --- |
| 1 | Climatic factors | Based on the meteorological observation data from 1950 to 2000, the resolution is 1km. | Mean precipitation in January (mm) | Prec1 | continuous | monthly mean precipitation from January to December | included |
| 2 |  |  | Mean precipitation in February (mm) | Prec2 | continuous |  | included |
| 3 |  |  | Mean precipitation in March (mm) | Prec3 | continuous |  | excluded |
| 4 |  |  | Mean precipitation in April (mm) | Prec4 | continuous |  | included |
| 5 |  |  | Mean precipitation in May (mm) | Prec5 | continuous |  | excluded |
| 6 |  |  | Mean precipitation in June (mm) | Prec6 | continuous |  | excluded |
| 7 |  |  | Mean precipitation in July (mm) | Prec7 | continuous |  | excluded |
| 8 |  |  | Mean precipitation in August (mm) | Prec8 | continuous |  | excluded |
| 9 |  |  | Mean precipitation in September (mm) | Prec9 | continuous |  | excluded |
| 10 |  |  | Mean precipitation in October (mm) | Prec10 | continuous |  | included |
| 11 |  |  | Mean precipitation in November (mm) | Prec11 | continuous |  | excluded |
| 12 |  |  | Mean precipitation in December (mm) | Prec12 | continuous |  | excluded |
| 13 |  |  | Mean precipitation in the growing season (mm) | Prec4_10 | continuous | mean precipitation from April to October | excluded |
| 14 |  |  | Mean temperature in January (℃×10) | Tmean1 | continuous | monthly mean temperature from January to December | excluded |
| 15 |  |  | Mean temperature in February (℃×10) | Tmean2 | continuous |  | excluded |
| 16 |  |  | Mean temperature in March (℃×10) | Tmean3 | continuous |  | included |
| 17 |  |  | Mean temperature in April (℃×10) | Tmean4 | continuous |  | excluded |
| 18 |  |  | Mean temperature in May (℃×10) | Tmean5 | continuous |  | excluded |
| 19 |  |  | Mean temperature in June (℃×10) | Tmean6 | continuous |  | excluded |
| 20 |  |  | Mean temperature in July (℃×10) | Tmean7 | continuous |  | excluded |
| 21 |  |  | Mean temperature in August (℃×10) | Tmean8 | continuous |  | excluded |
| 22 |  |  | Mean temperature in September (℃×10) | Tmean9 | continuous |  | excluded |
| 23 |  |  | Mean temperature in October (℃×10) | Tmean10 | continuous |  | excluded |
| 24 |  |  | mean temperature in November (℃×10) | Tmean11 | continuous |  | excluded |
| 25 |  |  | Mean temperature in December (℃×10) | Tmean12 | continuous |  | excluded |
| 26 |  |  | Mean temperature in the growing season (℃×10) | Tmean4_10 | continuous | mean temperature from April to October | included |
| 27 |  |  | Mean sunshine duration in January (h×10) | Smean1 | continuous | monthly sunshine duration from January to December | excluded |
| 28 |  |  | Mean sunshine duration in February (h×10) | Smean2 | continuous |  | excluded |
| 29 |  |  | Mean sunshine duration in March (h×10) | Smean3 | continuous |  | excluded |
| 30 |  |  | Mean sunshine duration in April (h×10) | Smean4 | continuous |  | excluded |
| 31 |  |  | Mean sunshine duration in May (h×10) | Smean5 | continuous |  | excluded |
| 32 |  |  | Mean sunshine duration in June (h×10) | Smean6 | continuous |  | excluded |
| 33 |  |  | Mean sunshine duration in July (h×10) | Smean7 | continuous |  | excluded |
| 34 |  |  | Mean sunshine duration in August (h×10) | Smean8 | continuous |  | excluded |
| 35 |  |  | Mean sunshine duration in September (h×10) | Smean9 | continuous |  | excluded |
| 36 |  |  | Mean sunshine duration in October (h×10) | Smean10 | continuous |  | excluded |
| 37 |  |  | Mean sunshine duration in November (h×10) | Smean11 | continuous |  | excluded |
| 38 |  |  | Mean sunshine duration in December (h×10) | Smean12 | continuous |  | excluded |
| 39 |  |  | Mean sunshine duration in the growing season (h×10) | Smean4_10 | continuous | mean sunshine duration from April to October | included |
| 40 |  |  | Mean annual precipitation (mm) | PrecAnnu | continuous | - | excluded |
| 41 |  |  | Mean annual temperature (℃×10) | TempAnnu | continuous | - | included |
| 42 |  |  | Mean annual sunshine duration (h×10) | SunshineAnnu | continuous | - | included |
| 43 |  |  | Monthly mean of temperature diﬀerence between day and night (℃×10) | TempDiﬀ | continuous | - | excluded |
| 44 |  |  | Standard deviation of seasonal changes in temperature | TempSeasonality | continuous | standard deviation of mean temperature in each season×100 (SD×100) | included |
| 45 |  |  | Range of mean annual temperature (℃×10) | TempRange | continuous | annual temperature diﬀerence between day and night | included |
| 46 |  |  | Seasonal precipitation variation coefficient | PrecVari | continuous | (standard deviation/mean) ×100% | excluded |
| 47 |  |  | Isothermality | - | continuous | (monthly mean of temperature diﬀerence between day and night/ range of mean annual temperature) ×100 | excluded |
| 48 |  |  | Precipitation of the warmest quarter (mm) | Bio1 | continuous | - | excluded |
| 49 |  |  | Precipitation of the coldest quarter (mm) | Bio2 | continuous | - | excluded |
| 50 |  |  | Precipitation of the driest quarter (mm) | Bio3 | continuous | - | excluded |
| 51 |  |  | Precipitation of the wettest quarter (mm) | Bio4 | continuous | - | excluded |
| 52 |  |  | Precipitation of the driest month (mm) | Bio5 | continuous | - | excluded |
| 53 |  |  | Precipitation of the wettest month (mm) | Bio6 | continuous | - | excluded |
| 54 |  |  | Mean temperature of the warmest quarter (℃×10) | Bio7 | continuous | - | excluded |
| 55 |  |  | Mean temperature of the coldest quarter (℃×10) | Bio8 | continuous | - | excluded |
| 56 |  |  | Mean temperature of the driest quarter (℃×10) | Bio9 | continuous | - | excluded |
| 57 |  |  | Mean temperature of the wettest quarter (℃×10) | Bio10 | continuous | - | excluded |
| 58 |  |  | Maximum temperature of the warmest month (℃×10) | Bio11 | continuous | - | excluded |
| 59 |  |  | Minimum temperature of the coldest month (℃×10) | Bio12 | continuous | - | excluded |
| 60 | Edaphic factors | Based on a 1:100,000 soil map of the People’s Republic of China (compiled in 1995) provided by the Second National Land Survey, FAO-90 is the soil classification system used | Soil organic carbon content (%) | SoilCarbon | continuous | soil organic carbon content (0–30 cm topsoil) | excluded |
| 61 |  |  | Soil texture | SoilTexture | categorical | - | included |
| 62 |  |  | Soil available water content level | SoilWater | categorical | - | included |
| 63 |  |  | Soil type | SoilType | categorical | based on FAO-90 | included |
| 64 |  |  | Cation exchange capacity of the soil (%) | SoilCation | continuous | cation exchange capacity of the soil (0–30 cm topsoil) | included |
| 65 |  |  | Soil pH | pH | continuous | - | included |
| 66 |  |  | Soil clay content (%) | SoilClay | continuous | soil clay content (0–30 cm topsoil) | excluded |
| 67 |  |  | Soil sand content (%) | SoilSand | continuous | soil sand content (0–30 cm topsoil) | included |
| 68 | Topographic factors | The resolution is 1km. | Slope (°) | - | continuous | - | included |
| 69 |  |  | Aspect | - | categorical | - | included |
| 70 |  |  | Altitude (m) | - | continuous | - | included |
| 71 | Biotic factors | Based on vegetation subtype data from a vegetation map of the People’s Republic of China (1:100,000) published by the Institute of Botany, Chinese Academy of Sciences. | Vegetation type | VegType | categorical | the sub-category of vegetation in the “Plant Map of the People’s Republic of China (1:1,000,000)” | included |
| 72 | Comprehensive meteorological indices | Derived from Kira’s thermal index and Xu’s modified version of Kira’s humidity index | Warm index (℃) | WI | continuous | - | excluded |
| 73 |  |  | Humid index (mm∙℃-1) | HI | continuous | - | excluded |
| 74 |  |  | Cold index (℃) | CI | continuous | - | excluded |

Appendix 2. Suitability level for A. membranaceus var. mongholicus distribution under various administrative levels

| **Leagues or cities** | **Optimum area** | **Suitable area and secondarily suitable area** | **Unsuitable area** |
| --- | --- | --- | --- |
| Bayannur League | central and eastern Urad Front Banner, and southeastern Urad Middle Banner | northern and western Urad Front Banner, central Urad Middle Banner, Wuyuan County, Linhe District, Hanggin Rear Banner, central and southern Dengkou County, and Urat Rear Banner | northwestern Urad Middle Banner, Urat Rear Banner |
| Baotou City | southeastern Darhan Muminggan Joint Banner, Guyang County, northern Tumd Right Banner, Hondlon District, Shiguai District, Jiuyuan District, and Donghe District | southern Tumd Right Banner, Bayan Obo Mining District, and central and northern Darhan Muminggan Joint Banner | - |
| Wulanchabu City | southern Siziwang Banner, central and northern Chahar Right Wing Middle Banner, Chahar Right Wing Back Banner, Chahar Right Wing Front Banner, Xinghe County, Shangdu County, Huade County, central Zhuozi County, and Feng Chin | central and northern Siziwang Banner, western and southern Chahar Right Wing Middle Banner, southwest Chahar Right Wing Back Banner and Chahar Right Wing Front Banner, southern Xinghe County, Langcheng County, and northwestern and southern Zhuozi County and Feng Chin | central Siziwang Banner |
| Chifeng City | central and southern Linxi County, Bairin Right Banner, Bairin Left Banner, Ar Horqin Banner, Ongniud Banner, central and northern Aohan Banner, central and eastern Songshan District, Yuanbaoshan District, Hongshan District, Harqin Banner, Hexigten Banner, and northeastern Ningcheng County | northern Linxi County, Bairin Left Banner, Ar Horqin Banner and Ongniud Banner, northwestern and southwestern Bairin Right Banner and Hexigten Banner, southern Aohan Banner, western Songshan District, Yuanbaoshan District, Hongshan District and Harqin Banner, and central and southern Ningcheng County | - |
| Tongliao City | southwestern Jarud Banner, and Nauman Banner | northern and southeastern Jarud Banner and Nauman Banner, Holingola, Horqin Left Wing Middle Banner, Kailu County, Horqin District, Kulun Banner, and northwestern Horqin Left Wing Rear Banner | central and southern Horqin Left Wing Rear Banner |
| Ordos City | Dalad Banner, Dongsheng District, northwestern Jungar Banner, eastern Hanggin Banner, Otog Banner, western Ejin Horo Banner, and Uxin Banner | southern Dongsheng District, central and western Hanggin Banner and Otog Banner, southern and eastern Ejin Horo Banner, Uxin Banner and Jungar Banner, and Otog Front Banner | northwestern Hanggin Banner |
| Huhhot City | Wuchuan County, central Tumd Left Banner, central and northern Togtoh County, northwestern Horinger, Yuquan District, and central and western Saihan District | southern Togtoh County, northeastern and southern Horinger, eastern Saihan District, southwestern Xicheng District, and Qingshuihe County | - |
| Xing'an League | central and southern Horqin Right Wing Middle Banner, Tuquan County, Horqin Right Wing Front Banner, and Ulanhot | northwestern and eastern Horqin Right Wing Middle Banner, southeastern Tuquan County, northern and eastern Horqin Right Wing Front Banner, and Jalaid Banner | Arxan |
| Xilin Gol | central and southern Xianghuang Banner, Zhengxiangbai Banner, Zhenglan Banner, Taibus Banner, and Duolun County | northern Xianghuang Banner, Zhengxiangbai Banner and Zhenglan Banner, central Sonid Right Banner, eastern and southern Sonid Left Banner, Abag Banner, Xilinhot, West Ujimqin Banner, and East Ujimqin Banner | northwestern Sonid Right Banner, northern Sonid Right Banner, Erenhot, northwestern West Ujimqin Banner, and northern East Ujimqin Banner |
| Hulunbeir City | northern Hulunbeir | southern, eastern, and northwestern Hulunbeir | northern and western Alashan, central and western Hulunbeir |
| Alashan | - | central and southern Alashan | northern Alashan |
| Wuhai City | - | Wuhai City | - |

Appendix 3. Calycosin-7-glucoside and astragaloside IV content in A. membranaceus var. mongholicus samples
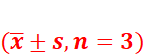


| **NO.** | **Content of Astragaloside IV (%)** | **Content of Calycosin-7 glucoside (%)** | **NO.** | **Content of Astragaloside IV (%)** | **Content of Calycosin-7 glucoside (%)** |
| --- | --- | --- | --- | --- | --- |
| S1 | 0.0420±0.0022 | 0.0340±0.0092 | S33 | 0.0611±0.0049 | 0.0370±0.0052 |
| S2 | 0.0522±0.0098 | 0.0413±0.0046 | S34 | 0.0790±0.0036 | 0.0240±0.0022 |
| S3 | 0.0530±0.0078 | 0.0200±0.0242 | S35 | 0.0955±0.0010 | 0.0654±0.0076 |
| S4 | 0.0410±0.0094 | 0.0951±0.0064 | S36 | 0.0920±0.0086 | 0.0610±0.0112 |
| S5 | 0.0472±0.0012 | 0.0382±0.0030 | S37 | 0.1180±0.0012 | 0.1030±0.0032 |
| S6 | 0.0221±0.0144 ˆ | 0.0249±0.0300 | S38 | 0.0428±0.0024 | 0.2390±0.0146 |
| S7 | 0.0840±0.0017 | 0.0537±0.0036 | S39 | 0.0585±0.0052 | 0.0324±0.0047 |
| S8 | 0.0840±0.0064 | 0.0527±0.0041 | S40 | 0.0497±0.0016 | 0.2330±0.0052 |
| S-9 | 0.0981±0.0056 | 0.1060±0.0012 | S41 | 0.0534±0.0057 | 0.1422±0.0045 |
| S10 | 0.0445±0.0043 | 0.0428±0.0032 | S42 | 0.0734±0.0092 | 0.0225±0.0046 |
| S11 | 0.1150±0.0021 | 0.0663±0.0074 | S43 | 0.0630±0.0041 | 0.0810±0.0032 |
| S12 | 0.1065±0.0040 | 0.0258±0.0044 | S44 | 0.0580±0.0018 | 0.0230±0.0010 |
| S13 | 0.0538±0.0049 | 0.0812±0.0084 | S45 | 0.0926±0.0054 | 0.2024±0.0202 |
| S14 | 0.0342±0.0016 ˆ | 0.0239±0.0061 | S46 | 0.0330±0.0021 ˆ | 0.0250±0.0038 |
| S15 | 0.0121±0.0030 ˆ | 0.0820±0.0241 | S47 | 0.0487±0.0081 | 0.0225±0.0026 |
| S16 | 0.0500±0.0012 | 0.0255±0.0023 | S48 | 0.0749±0.0052 | 0.0543±0.0155 |
| S17 | 0.0417±0.0092 | 0.1315±0.0132 | S49 | 0.0562±0.0033 | 0.0777±0.0024 |
| S18 | 0.0578±0.0016 | 0.0456±0.0046 | S50 | 0.0762±0.0014 | 0.0697±0.0100 |
| S19 | 0.0571±0.0051 | 0.0449±0.0072 | S51 | 0.0517±0.0085 | 0.0546±0.0036 |
| S20 | 0.0844±0.0018 | 0.1118±0.0301 | S52 | 0.0890±0.0023 | 0.0770±0.0020 |
| S21 | 0.0848±0.0033 | 0.0813±0.0046 | S53 | 0.0637±0.0094 | 0.0502±0.0082 |
| S22 | 0.0499±0.0092 | 0.1503±0.0112 | S54 | 0.0678±0.0048 | 0.0464±0.0038 |
| S23 | 0.0761±0.0140 | 0.0792±0.0072 | S55 | 0.0094±0.0021 ˆ | 0.0464±0.0142 |
| S24 | 0.0601±0.0023 | 0.0992±0.0012 | S56 | 0.1782±0.0063 | 0.1186±0.0055 |
| S25 | 0.0480±0.0085 | 0.0422±0.0088 | S57 | 0.0107±0.0074 ˆ | 0.0463±0.0074 |
| S26 | 0.0463±0.0043 | 0.0491±0.0080 | S58 | 0.0423±0.0015 | 0.0652±0.0078 |
| S-27 | 0.0575±0.0055 | 0.0615±0.0082 | S59 | 0.1338±0.0013 | 0.0235±0.0052 |
| S-28 | 0.0550±0.0074 | 0.0643±0.0090 | S60 | 0.0647±0.0016 | 0.0236±0.0066 |
| S-29 | 0.0910±0.0089 | 0.0790±0.0062 | S61 | 0.0660±0.0085 | 0.0673±0.0044 |
| S-30 | 0.0468±0.0048 | 0.0780±0.0082 | S62 | 0.0550±0.0044 | 0.0240±0.0084 |
| S-31 | 0.0910±0.0014 | 0.0350±0.0105 | S63 | 0.0510±0.0043 | 0.083±0.0090 |
| S-32 | 0.0393±0.0063ˆ | 0.0678±0.0050 |  |  |  |

Note: The 2015 edition of *Chinese Pharmacopoeia* stipulates that this product shall contain not less than 0.040% astragaloside IV and not less than 0.020% calycosin-7-glucoside. “ˆ” indicates that astragaloside IV did not reach the limit standard of Chinese Pharmacopoeia.

Appendix 4. Geographic information of A. membranaceus var. mongholicus samples

| **NO.** | **Collection site** | **latitude** | **longitude** |
| --- | --- | --- | --- |
| S1 | Shiyifenzi village of Ming'an town in Urad Front Banner, Bayan Nur | 40°54′37.75″ | 109°48′53.45″ |
| S2 | Gonghudong Gacha of E'erdengbulage Sumu (in English: counties) in Urad Front Banner, Bayan Nur | 40°51′21.58″ | 109°00′05.35″ |
| S3 | Yushuta village of Chaoyang town in Urad Front Banner, Bayan Nur | 40°54′56.31″ | 109°51′40.41″ |
| S4 | Yinjiangyaozi village of Chaoyang town in Urad Front Banner, Bayan Nur | 40°54′32.77″ | 109°48′59.55″ |
| S5 | Baiyanhua village of Xianfeng town in Urad Front Banner, Bayan Nur | 40°38′32.75″ | 109°12′48.27″ |
| S6 | Menghan village of Bayinbaolige town in Urad Back Banner, Bayan Nur | 41°33′57.85″ | 106°29′25.78″ |
| S7 | Qianheishahe village of Guyang County in Baotou | 40°38′50.86″ | 109°58′03.10″ |
| S8 | Xiaoyingtu village of Xiashihao town in Guyang County, Baotou | 40°54′09.15″ | 110°31′18.10″ |
| S-9 | Xiaobanglang village of Xiashihao town in Guyang County, Baotou | 41°00′30.42″ | 110°03′00.69″ |
| S10 | Qiandian village of Xiashihao town in Guyang County, Baotou | 40°54′28.07″ | 110°32′59.87″ |
| S11 | Zhuhuandi village of Xingshunxi town in Guyang County, Baotou | 41°16′29.25″ | 109°53′35.23″ |
| S12 | Shibutai village of Guyang County in Baotou | 40°54′41.49″ | 110°35′51.36″ |
| S13 | Beiwuta village of Yinhao town in Guyang County, Baotou | 41°11′47.62″ | 110°20′13.93″ |
| S14 | Nianfangdadui village of Yinhao town in Guyang County, Baotou | 41°06′42.60″ | 110°19′32.79″ |
| S15 | Huaishuo village of Huaishuo town in Guyang County, Baotou | 41°17′24.36″ | 110°18′29.96″ |
| S16 | Saihudong village of Wukehudong town in Darhan Muminggan Joint Banner, Baotou | 41°21′01.04″ | 110°31′20.63″ |
| S17 | Shuilongtan village of Wukehudong town in Darhan Muminggan Joint Banner, Baotou | 41°25′24.72″ | 110°32′10.07″ |
| S18 | Shibao village of Shibao town in Darhan Muminggan Joint Banner, Baotou | 41°22′03.80″ | 110°56′43.05″ |
| S19 | Bulang village of Xiaowengong town in Darhan Muminggan Joint Banner, Baotou | 41°33′08.88″ | 111°14′22.04″ |
| S20 | Daqian village of Wudangzhao town in Shiguai District, Baotou | 40°41′46.89″ | 110°20′38.34″ |
| S21 | Haizi village of Haizi town in Tumd Right Banner, Baotou | 40°24′44.84″ | 110°39′25.78″ |
| S22 | Subogai village of Subogai town in Tumd Right Banner, Baotou | 40°35′41.45″ | 110°40′39.95″ |
| S23 | Huanghuatan village of Wuchuan County in Huhhot | 40°59′49.19″ | 110°54′35.47″ |
| S24 | Yangchengshangou village of Wuchuan County in Huhhot | 41°12′20.16″ | 111°24′34.72″ |
| S25 | Pugesu village of Hale town in Wuchuan County, Huhhot | 41°13′40.23″ | 111°40′39.06″ |
| S26 | Xihongshan village of Wuchuan County in Huhhot | 41°11′38.14″ | 110°40′35.69″ |
| S27 | Halaheshao village of Wuchuan County in Huhhot | 40°58′21.42″ | 110°54′58.28″ |
| S28 | Hexiyao village of Halaheshao town in Wuchuan County, Huhhot | 40°56′56.04″ | 110°40′59.79″ |
| S29 | Shuangyucheng village of Erfenzi town in Wuchuan County, Huhhot | 41°18′55.86″ | 110°56′51.23″ |
| S30 | Maolinba village of Deshenggou Country in Wuchuan County, Huhhot | 40°56′23.12″ | 111°14′27.20″ |
| S31 | Qianyaozi village of Deshenggou Country in Wuchuan County, Huhhot | 40°57′45.12″ | 111°04′38.54″ |
| S32 | Jiuguan village of Deshenggou Country in Wuchuan County, Huhhot | 40°59′51.76″ | 111°09′09.20″ |
| S33 | Erdaohe Village of Yuquan District in Hohhot | 40°46′00.60″ | 111°35′44.77″ |
| S34 | Maqungou village of Chenguan town in Horinger County, Huhhot | 40°16′05.49″ | 111°43′01.38″ |
| S35 | Chengguai village of Shuanghe town in tuoketuo County, Huhhot | 40°17′23.22″ | 111°11′06.66″ |
| S36 | Jingxin Agriculture and Forestry Technology Co., Ltd. in tuoketuo County, Huhhot | 40°46′13.69″ | 111°42′33.29″ |
| S37 | Dianzi village of Dianzi town in Xinghe County, Ulanqab | 40°39′26.71″ | 113°56′07.38″ |
| S38 | Heitutai town in Feng Chin, Ulanqab | 40°33′28.48″ | 113°24′13.19″ |
| S39 | Guanzhuangzi village of Ulan Huar town in Siziwang Banner, Ulanqab | 41°37′0.92″ | 111°46′46.09″ |
| S40 | Huangyangcheng town in Chahar Right Middle Banner, Ulanqab | 41°19′01.06″ | 112°30′0.42″ |
| S41 | Dafangzi village of Xile town in Chahar Right Back Banner, Ulanqab | 41°16′24.00″ | 112°55′40.80″ |
| S42 | Shibaqing village of Shangdu County in Ulanqab | 41°34′03.00″ | 113°51′55.26″ |
| S43 | Yuzhou village of Bolihujing town in Shangdu County, Ulanqab | 41°47′45.65″ | 113°41′23.31″ |
| S44 | Ergudi village of Dakulun town in Shangdu County, Ulanqab | 41°58′33.12″ | 113°29′14.36″ |
| S45 | Shelin Gacha of Wulanhada town in Ulanhot, Hinggan League | 46°05′22.17″ | 122°07′22.43″ |
| S46 | Tarigenaobao Gacha of Samai Sumu in East WuZhu MuQinQi, Xilingol League | 45°59′22.06″ | 117°38′16.52″ |
| S47 | Yijiahe village of Duolun County in Xilingol League | 42°21′37.15″ | 116°34′48.86″ |
| S48 | Heishanzu village of Dahekou town in Duolun County, Xilingol League | 42°17′18.93″ | 116°45′45.31″ |
| S49 | Baiyinpuluo village of Caimushan town in Duolun County, Xilingol League | 42°15′18.03″ | 116°32′16.38″ |
| S50 | Baichengzi village of Caimushan town in Duolun County, Xilingol League | 42°17′23.89″ | 116°28′09.71″ |
| S51 | Wulan Gacha of Naritu Sumu of in ZhengLan flag, Xilingol League | 42°52′09.90″ | 115°48′42.67″ |
| S52 | Heichengzi twon of ZhengLan flag in Xilingol League | 42°00′50.78″ | 115°53′16.51″ |
| S53 | Lindong town of Balin left Banner in Chifeng | 44°36′01.49″ | 119°37′02.56″ |
| S54 | Haiyao village of Sanshan town in Balin right Banner, Chifeng | 44°04′36.73″ | 120°13′54.38″ |
| S55 | Niuyingzi village of Niujiayingzi town in Kalaqin Banner, Chifeng | 42°05′59.07″ | 118°47′26.07″ |
| S56 | Dongyingzi village of Xiaochengzi town Ningcheng County, Chifeng | 41°46′59.07″ | 119°01′0.03″ |
| S57 | Qiaotou town of Weng Niute Banner in Chifeng | 42°36′26.96″ | 118°54′34.75″ |
| S58 | Sanjiazi village of Tuchengzi town in Naiman Banner, Tongliao | 42°21′06.52″ | 120°48′10.48″ |
| S59 | Kangmei Pharmaceutical Co., Ltd. of Desheng village in Naiman Banner, Tongliao | 43°21′07.62″ | 121°19′57.83″ |
| S60 | Deshengtun village of Dongming town in Naiman Banner, Tongliao | 43°18′52.37″ | 121°21′47.70″ |
| S61 | Bailu island of Eerguna City in Hulun Buir | 51°56′44.59″ | 120°51′40.48″ |
| S62 | Jianguo village of Arun Banner in Hulun Buir | 48°07′12.41″ | 123°26′48.96″ |
| S63 | Alihe twon of Oroqen Autonomous Banner in Hulun Buir | 50°35′19.71″ | 123°43′08.09″ |

Note: “S” stands for sampling point of *A. membranaceus* var. *mongholicus*

1. ^*^ Corresponding authors

   Li MH Tel: +86-472-716 7739, E-mail: [prof_liminhui@yeah.net](mailto:prof_liminhui@yeah.net); Zhang CH Tel: +86-472-716 7795, E-mail: [zchlhh@126.com](mailto:zchlhh@126.com) [↑](#footnote-ref-2)
